# Development and in vitro characterization of a humanized scFv against fungal infections

**DOI:** 10.1371/journal.pone.0276786

**Published:** 2022-10-31

**Authors:** Tomas Di Mambro, Tania Vanzolini, Marzia Bianchi, Rita Crinelli, Barbara Canonico, Filippo Tasini, Michele Menotta, Mauro Magnani

**Affiliations:** 1 Diatheva s.r.l., Cartoceto, Italy; 2 Department of Biomolecular Sciences, University of Urbino Carlo Bo, Urbino, Italy; Weizmann Institute of Science, ISRAEL

## Abstract

The resistance and the birth of new intrinsic and multidrug-resistant pathogenic species like *C*. *auris* is creating great concern in the antifungal world. Given the limited drug arsenal and the lack of effectiveness of the available compounds, there is an urgent need for innovative approaches. The murine mAb 2G8 was humanized and engineered *in silico* to develop a single-chain fragment variable (hscFv) antibody against β-1,3-glucans which was then expressed in *E*. *coli*. Among the recombinant proteins developed, a soluble candidate with high stability and affinity was obtained. This selected protein is VL-linker-VH oriented, and it is characterized by the presence of two ubiquitin monomers at the N-terminus and a His tag at the C-terminus. This construct, Ub_2_-hscFv-His, guaranteed stability, solubility, efficient purification and satisfactory recovery of the recombinant product. HscFv can bind β-1,3-glucans both as coated antigens and on *C*. *auris* and *C*. *albicans* cells similarly to its murine parental and showed long stability and retention of binding ability when stored at 4°, -20° and -80° C. Furthermore, it was efficient in enhancing the antifungal activity of drugs caspofungin and amphotericin B against *C*. *auris*. The use of biological drugs as antifungals is limited; here we present a promising hscFv which has the potential to be useful in combination with currently available antifungal drugs.

## Introduction

The use of monoclonal antibodies as therapeutics has met a rapid evolution mainly due to their computational identification and/or design combined with efficient and rapid screening methods [1]. In 1986 the FDA approved the first monoclonal antibody, Muromonab-CD3, a murine antibody anti-CD3 used for the prevention of transplant rejection [2]. Its murine nature turned out to be a great obstacle as it stimulated the immune system giving severe immunogenicity reactions [3]. From that moment humanization processes began, and a few years later Natalizumab, a humanized monoclonal antibody anti-α4β1 integrin for the treatment of multiple sclerosis, reached the market [4]. Almost simultaneously, advances in recombinant antibody technologies promoted the manipulation of antibody fragments, allowing research, diagnoses, and therapies to benefit from several engineered products [5–10]. The first chimeric antigen-binding fragment (Fab) anti-GPIIb/IIIa Abciximab was approved by the FDA in 1994 for the inhibition of platelet aggregation in cardiovascular diseases [11]. Among the antibody fragments, the single-chain fragment variable (scFv), represents an intriguing format as it consists of only the variable regions of the heavy and light chains. Their greater penetration and facilitated access to the antigen given by their smaller sizes, together with the lack of glycosylation, the better pharmacokinetic profile, the lower immunogenicity risk due to the absence of the Fc domain and the prospect of producing high quantities at low costs are great advantages. However, the rapid clearance, the low stability, the risk of aggregation and the decreased binding affinity compared to the parental full-length protein have heavily limited their entry into the market. Mycograb (Efungumab) is a clear example of these difficulties [7]. Mycograb is a human-derived scFv targeting fungal heat shock protein 90 (HSP90) and a potential antifungal agent for the treatment of invasive candidiasis that reached clinical trials [12, 13]. However, it never received marketing authorization mainly because of its tendency towards aggregation in solution and other safety issues [14]. Despite the difficulties in scFv development, we aimed to produce an innovative humanized scFv (hscFv) to be used in fungal infections. Nowadays there is an urgent need for new antifungal drugs able to target well-known and new pathogenic fungi. Indeed, there is rising concern particularly for intrinsic and multidrug resistance found for example in *C*. *auris*. To develop our hscFv we started from 2G8, a murine monoclonal IgG2b antibody able to selectively bind β-1,3-glucans, which are fundamental components of the fungal cell wall, hence effective targets of several pathogenic fungi [15–18]. Considering the well-known properties of ubiquitin and ubiquitin-like proteins (e.g. SUMO) as fast folding soluble polypeptides, and their wide use as fusion partners [19, 20], a new expression strategy based on the ubiquitin/histidine combinatorial tagging was developed. In literature ubiquitin proved efficient in improving the solubility and the proper folding of the fused proteins but also conferred protection from proteolytic degradation [21, 22]. These unique features encouraged its use as fusion partner to increase recombinant protein solubility in combination with affinity tags (such as the His tag) for subsequent rapid purification [23]. In our case, we obtained a soluble, stable, and pure hscFv. In addition, based on our previous results with H5K1 (the humanized full-length antibody derived from 2G8) [24], we assessed its binding activity not only on coated antigen laminarin but also on *C*. *auris* and *C*. *albicans* cells. Finally, seeing the promising results achieved by combining H5K1 with commercially available antifungal drugs, we tested our hscFv both alone and in drug combinations against *C*. *auris* (selected as fungal model).

## Materials and methods

### Cell lines

In our studies we used *E*. *coli* BL21(DE3) (69450 Sigma-Aldrich), *C*. *auris* (DMS 21092) and *C*. *albicans* (ATCC 10231).

### Humanization of VH and VL regions of the murine monoclonal antibody 2G8

The amino acid sequence of the murine scFv was analysed by ExPasy [25] to evaluate the *instability index*. The murine variable regions (VH and VL) were compared with the murine databases IMGT mouse V genes, IMGT mouse D genes, and IMGT mouse J genes in *IgBlastTool* [26] software and were mutated following the germlines found. The VH and VL regions were humanized by *IgBlastTool* launching their nucleotide sequences against the human germline databases IMGT human V genes, IMGT human D genes and IMGT human J genes [27, 28]. According to the CDR-grafting technique, two strategies have been examined: the first one was based on the analysis of the whole murine VH and VL sequences while the second strategy considered just the single frameworks. In both cases, the germlines with the highest identity with the initial sequences were selected and non-homologous amino acids were changed only in framework regions (FRW). The two humanized scFvs (hscFv) were assembled and their *instability indexes* were analyzed once again by ExPasy. The chosen variable regions were checked separately in PDB databases [29] and the output sequences with the closest homology and with an X-ray crystallographic resolution ≤ 2 Å were selected. In order to contain the reduction of binding affinity and specificity, the amino acids in the Vernier Zone positions [30] were back-mutated to the original murine ones.

### Humanized scFvs (hscFv) in VH-linker-VL and VL-linker-VH orientations: Construction and cloning in pET22b(+)

The hscFv oriented in VH-linker-VL was synthesized *de novo* by GenScript and optimized with the codon usage for the expression in *E*. *coli* BL21(DE3) strain. The variable domains, connected by a 15 amino acid linker (G_4_S)_3,_ were cloned between NcoI and HindIII restriction sites into the pET22b(+) expression vector with a C-terminal 6-Histidine tag (His tag) for affinity purification (VH-linker-VL-His, S1 Fig). To obtain the construct in reverse orientation (VL-linker-VH-His, S1 Fig), the variable regions were amplified separately by endpoint PCR with specific primers (Table 1) using the high-fidelity thermophilic DNA polymerase Vent DNA Polymerase (BioLabs). After purification with MiniElute PCR purification Kit (Qiagen), 50 ng of each purified variable region were used to create the new hscFv by SOE-PCR. The thermal profile of the reaction was: denaturation at 95°C for 3 min, 5 cycles of denaturation at 95°C for 1 min, annealing at 63°C for 1 min and extension at 72°C for 1 min, 5 cycles of denaturation at 95°C for 1 min, annealing at 56°C for 30 sec and extension at 72°C for 1 min, 25 cycles of denaturation at 95°C for 30 sec and extension at 72°C for 1 min. The resulting fragment was purified with the MiniElute Gel-extraction purification Kit (Qiagen) and digested with NcoI and HindIII restriction enzymes, as for the expression vector pET22b(+). The ligase reaction was performed with Anza Ligase (ThermoFisher) enzyme and competent *E*. *coli* BL21(DE3) cells were transformed. The positive clones were checked by colony PCR and underwent a miniprep with QiaPrep Spin Miniprep Kit (Qiagen) to be sequenced. T7 promoter and T7 terminator primers were used for both colony PCR and sequencing.

**Table 1:** Primers used to amplify VL-linker-VH sequence.

*Sequence (5’>3’)*	*F/R*
5’-TTCCTGCCATGGACATTGTGATGACCCAGAC-3’	VL-F
5’-ACCAGAGCCGCCGCCGCCGCTACCACCACCACCACGTTTGATTTCCACTTTGG-3’	VL-R
5’-AGCGGCGGCGGCGGCTCTGGTGGTGGTGGTTCCCAGGTTCAACTGGTCCAAAG-3’	VH-F
5’-GGAAGTTAAGCTTTTAGGAACTAACGGTCACCAGG-3’	VH-R

F: forward, R: reverse.

### Cloning of the human ubiquitin coding sequence into the pET45b(+)

The His-tagged ubiquitin-based expression vectors were constructed starting from the pET45b(+) (Novagen) plasmid backbone. The pET45b(+) was digested with PmlI and BamHI to insert the coding sequence for one or more ubiquitin (Ub) monomers, in frame with the N-terminal His tag, provided by the vector itself. The wild-type Ub coding sequence was PCR amplified from HeLa cDNA, obtained from total RNA, purified with the RNeasy Plus Mini kit (Qiagen) and reverse-transcribed using PrimeScript^TM^ RT Master Mix (Takara Bio), according to manufacturer´s instructions. Amplification of the Ub coding sequence was performed with the Platinum Pfx DNA polymerase (Invitrogen), according to the protocol, and the degenerate primers reported in Table 2.

**Table 2:** Primers used to amplify the ubiquitin coding sequence.

*Sequence (5’>3’)*	*F/R*
5’-CGTCACGTCACGTGATGCAGATCTTCGTGAAGACC-3’	F
5’- ACGTGACGGGATCCACCGCGGAGACGGAGCACCAGGTGC-3’	R

F: forward, R: reverse.

The forward primer was engineered to be cut with PmlI restriction enzyme, while the reverse primer, carrying a BamHI cutting site, was designed to allow the deletion of the translation stop codon at the end of the insert. Moreover, the presence of a SacII cutting site in the reverse primer which matches with the last Ub codons could be exploited to clone downstream any coding sequence hence, to obtain a fusion product with Ub at the N-terminus without any intervening amino acid residue and with an available internal SacII cleavage site. The PCR conditions were: 2 min at 94°C; 35 cycles of denaturation at 94°C for 15 s, annealing at 62°C for 15 s, and extension at 68°C for 1 min. At the end of PCR cycles, the amplified products were analyzed through agarose gel electrophoresis. Ub is encoded in mammals by different genes which are transcribed by different mRNAs containing one (UBA52 and RPS27A) or more Ub CDS in tandem (3 for UBB and 9 for UBC, respectively) sharing a high homology. This explains the PCR output with the two degenerate primers reported in Table 2, which displayed multiple bands corresponding to one or more Ub CDS. The PCR products corresponding to the Ub monomer (Ub_1_), dimer (Ub_2_) and trimer (Ub_3_) were gel-purified with the Gel Extraction Kit, double-digested with PmlI and BamHI and then inserted into the pET45b(+) vector cut with the same restriction enzymes (His-Ub_1_, His-Ub_2_ and His-Ub_3_ respectively, S1 Fig). Screening and purification of positive clones were performed as above. All pET45b(+)/Ub constructs were confirmed through DNA sequencing using a PE310 Perkin Elmer capillary sequencer.

### Subcloning of the VL-linker-VH oriented-hscFv into the Ub-engineered pET45b(+) vectors and of the Ub_2_-hscFv and Ub_3_-hscFv coding sequences into pET22b(+)

The VL-linker-VH oriented hscFv was amplified from the pET22b(+) vector with specific primers and purified with the MinElute PCR Purification Kit. Based on the target cloning vector, the PCR product was digested with different restriction enzymes: PmlI (Anza) and BamHI (Anza) to subclone it into the pET45b(+) downstream the 6-His tag and KspI (Roche, isoschizomer of SacII) and HindIII (Anza) to introduce it into the pET45b(+) as it is (His-hscFv, S1 Fig) or downstream the Ub-trimer/dimer/monomer CDS (His-Ub_1_-hscFv, His-Ub_2_-hscFv and His-Ub_3_-hscFv respectively, S1 Fig). After purification with the MinElute PCR Purification Kit, the digested hscFv insert was ligated with each of the mentioned plasmids already digested with the corresponding restriction enzymes and treated with Calf-intestinal alkaline phosphatase (Anza). Competent *E*. *coli* BL21(DE3) cells were transformed with the ligase reactions and the colonies were checked by colony PCR. The positive clones were used for plasmid miniprep and then sequenced. To obtain the C-terminal His-tagged Ub_2_- and Ub_3_-hscFv fusion products (Ub_2_-hscFv-His and Ub_3_-hscFv-His respectively, S1 Fig), the expression cassettes Ub_2_-hscFv and Ub_3_-hscFv were amplified from the Ub-modified pET45b(+) expression vector with specific primers containing NdeI and HindIII restriction sites. The amplified DNA fragments were purified with the Qiagen PCR purification kit and treated with NdeI and HindIII. The same enzymes were designed to remove the pel B sequence from the pET22b(+) plasmid before ligation with the digested inserts. Ligation products were transformed into competent *E*. *coli* BL21(DE3) cells. Bacteria were streaked on agarose plates containing 100 μg/ml ampicillin (Sigma-Aldrich) and the vectors with the right inserts were selected and used for expression.

### Expression of recombinant proteins

*E*. *coli* BL21(DE3) cells transformed with the selected plasmids were inoculated overnight at 30°C in Luria-Bertani (LB) medium (10 g/L Trypton, 5 g/L Yeast Extract, 10 g/L NaCl, pH 7.5) and supplemented with ampicillin 100 μg/ml (A_100_). The following day, the cell culture was diluted in fresh LB medium+A_100_ to an optical density of 0.1 and left to grow at 25°C until the OD_600nm_ reached an absorbance of 0.6/ 0.8. An aliquot of the culture was then removed and used as Not-Induced (NI) control whilst 0.5 mM isopropylthio-β-D-galactopyranoside (IPTG) (Sigma-Aldrich) was added to the remaining culture volume to induce the expression of the recombinant protein. The expression was carried out at 25°C for three hours with withdrawals and OD_600nm_ measurements at every hour (the remaining volume was often used for the purification processes). The harvested aliquots were pelleted by centrifugation at 4°C for 20 minutes at 3,000 rpm, suspended in 0.5 ml of lysis buffer (Na/K phosphate buffer 20 mM, glycerol 10% (v/v), NaCl 0.3 M, β-mercaptoethanol (BME) 3 mM, PMSF 1 mM, pH 7.4) and sonicated 3 times (Ultrasonic Cell Crusher 60 W, 30 sec on ice). Extracts were centrifuged at 4°C for 20 minutes at 12,000 rpm and the supernatants, representing the soluble fractions, were separated from the pellets. The latter were resuspended in 0.5 ml of denaturing buffer (Na/K phosphate buffer 20 mM, urea 8 M, glycerol 10% (v/v), NaCl 0.3 M, BME 10 mM, pH 8.0). After one cycle of sonication, the suspensions were rotated for one hour and then centrifuged for 20 minutes at RT at 12,-000 rpm. The supernatants obtained represented the insoluble fraction (inclusion bodies (IBs)). Protein concentration of soluble and insoluble fractions was measured by the Bradford assay (Bio-Rad), using bovine albumin as reference standard, and proteins were analyzed by SDS-PAGE.

### Purification of recombinant proteins by IMAC

The *E*. *coli* pellet was suspended in lysis buffer (Na/K phosphate buffer 20 mM, glycerol 10% (v/v), NaCl 0.3 M, BME 3 mM, PMSF 1 mM, pH 7.4), and cell disruption was obtained by using a French pressure cell press (Avastin, Emulsiflex B15) operating at pressures between 10,000 and 13,000 Psi. The cell extract was then centrifuged at 12,000 rpm for 30 min. at 4°C to separate the soluble and insoluble fractions (IBs). Proteins were purified by Immobilized metal ion affinity chromatography (IMAC) using an AKTA purifier chromatography system (GE Healthcare). The soluble fraction was directly loaded onto a 5 ml-HisTrap HP column (GE Healthcare, Bucks, UK) equilibrated with 5 CV (column volume) of loading buffer (Na/K phosphate buffer 20 mM, glycerol 10% (v/v), NaCl 0.3 M, BME 3 mM, pH 7.4). The column was washed with 20 CV of loading buffer and the elution was performed with 5 CV of the same buffer supplemented with 50, 250 and 500 mM of imidazole in sequence (Sigma-Aldrich).

The insoluble fraction was suspended in denaturing buffer (Na/K phosphate buffer 20 mM, urea 8 M, glycerol 10% (v/v), NaCl 0.3 M, BME 10 mM and 30 mM of imidazole, pH 8.0) and incubated at RT for 60 min. The sample was centrifuged at 12,000 rpm for 30 min and, the urea supernatant collected and loaded onto the HisTrap HP-column equilibrated with 5 CV loading buffer (Na/K phosphate buffer 20 mM, urea 8 M, NaCl 0.3 M, glycerol 10% (v/v), BME 10 mM, imidazole 30 mM, pH 8.0). After sample loading, the column was washed with 20 CV of loading buffer and the protein was refolded on column by a urea linear gradient of 30 CV from 8 M to 0.5 M. The protein was eluted with 5 CV of 50, 250 and 500 mM of imidazole in 20 mM of phosphate buffer, 0.5 M urea, 0.3 M NaCl, 10% (v/v) glycerol, 3 mM BME, pH 8.0.

### Optimization of the purification protocol for the isolation of the Ub_2_-hscFv-His protein from the soluble fraction

The first optimization step involved the shift of the pH to a more basic value, i.e. 8.5 to improve protein binding to the Ni^2+^ ligand. To achieve this, the phosphate buffer was replaced with 20 mM Tris-HCl pH 8.5 in all buffer solutions used for the purification step. The *E*. *coli* pellet was suspended in 25 ml of Tris lysis buffer, disrupted by French Press and centrifugated. The soluble fraction was loaded into a 30 ml bed volume of Ni^2+^ Sepharose HP (GE Healthcare, Bucks, UK). The second step of optimization was developed with the aim of reaching a higher level of purity. Therefore, after sample loading and column equilibration, 5 CV of loading buffer containing 100 mM imidazole were applied to the column before elution. To further improve the hscFv purity, the third step of optimization required the addition of a negative passage into Q Sepharose FF column (GE Healthcare, Bucks, UK) before IMAC. After cell lysis, the soluble fraction was loaded onto a Q Sepharose FF column previously equilibrated with 5 CV of Tris-HCl loading buffer. The column was then washed with 20 CV of loading buffer. The flow-through fractions containing the protein were combined, concentrated and, pH and NaCl were adjusted to 8.5 and 0.3M respectively, before loading onto the Ni^2+^ Sepharose HP column. The Ub_2_- hscFv-His protein was eluted from the column after application of an imidazole gradient from 100 to 250 mM (5CV). The elution fractions were analyzed by SDS-PAGE and the fractions containing the Ub_2_-hscFv-His protein (thereafter referred as to hscFv) were combined, concentrated, dialyzed to remove imidazole, and used to perform different analyses.

### SDS-PAGE and western immunoblotting analysis

Samples were diluted in SDS sample buffer supplemented with 4% (v/v) BME, vortexed and heated at 100°C for 3 min. Proteins were resolved onto SDS-PAGE polyacrylamide gels and visualized by gel staining with Brilliant Blue Coomassie R-250. For immunodetection the gels were electroblotted onto a nitrocellulose membrane (0.2 mm pore size) (Bio‐Rad) for 1 h at 100 V. The membranes were then blocked with 3% BSA (Bovine Serum Albumin, Sigma-Aldrich) in Tris buffer saline (TBS) + Tween 20 (Sigma-Aldrich) 0.1% for 1 h at RT and stained with an anti-6X His tag polyclonal antibody (OriGene). Detection was performed with horseradish peroxidase (HRP)‐conjugated secondary antibody (Bio‐Rad) and the enhanced chemiluminescence detection kit WesternBright ECL (Advansta) in a ChemiDoc MP Imaging System (Bio‐Rad).

### ELISA assay

A 96-well plate was coated with 50 μg/ml Laminarin (Sigma-Aldrich, L9634) in 0.05 M carbonate buffer, pH 9.6 overnight at 4°C. After blocking with BSA, different concentrations of hscFv (from 50 μg/ml to 0.003 μg/ml) were dispensed into the wells and incubated for 2 h at 37°C. Then, 100 μL/well of freshly prepared anti-6X His tag polyclonal antibody (OriGene) diluted in blocking solution (1:500) were added and the plates were incubated at 37°C for 1 h. Detection was obtained with goat anti-rabbit HRP secondary antibody and ABTS substrate (Roche Diagnostics). Absorbance at 405 nm was measured in a microplate reader (Bio-Rad).

### Mass spectrometry

Following the method of Zhang et al. [31], 10 pmol/μl of hscFv diluted in a 50/50 solution of H_2_O and acetonitrile supplemented with formic acid 0.1% were injected for direct infusion into the Orbitrap Exploris 240 Mass Spectrometer (Thermo Fisher Scientific) equipped with electrospray ionization source. The analysis was performed with a resolution of 15,000 at RT with an elution flow rate of 10 μl/min, in intact protein mode and the source-induced dissociation (SID) was set at 60 V without fragmenting the hscFv. The resulting spectrum was the average of all the scans acquired in the mass range from 600 to 1,400 mass-to-charge ratio (m/z) and deconvoluted with FreeStyle software (Thermo Fisher Scientific). The analysis was performed in-house (University of Urbino Carlo Bo–Department of Biomolecular Sciences—Biochemistry and Biotechnology section).

### Stability test

The purified hscFv was dialyzed to replace BME with 1,4-Dithiothreitol (DTT) as a stronger and more stable reducing agent, hence preferred for storage. The hscFv solution was divided in aliquots and stored at different temperatures for different time points (Table 3). The evaluation of the structural stability of the hscFv was performed through SDS-PAGE and western blot, while IC_50_ resulting from ELISA tests were compared to assess its activity.

**Table 3:** Schematic representation of the temperatures and time points for the stability test.

Temperatures	37°C	4°C	-20°C	-80°C
**Time points**	3 days	3 days		
	1 week	1 week		
	2 weeks	2 weeks		
	3 weeks	3 weeks		
		1 month	1 month	
		2 months	2 months	
		3 months	3 months	3 months

The hscFv stored at different temperatures was evaluated for its stability and activity in SDS-PAGE, western immunoblotting and ELISA assay after the reported timepoints.

### Immunofluorescence and flow cytometry

*Candida albicans* and *Candida auris* were inoculated overnight in RPMI+MOPS (0.165 M pH 7, Sigma-Aldrich). In order to investigate *C*. *albicans* in both hyphal and yeast form, two inocula for this specie were prepared, one in RPMI+MOPS (0.165 M pH 7) and the other in RPMI+MOPS (0.165 M pH 7) supplemented with 10% serum [32]. Inocula were washed and ~10^6^ CFU/ml were resuspended in PBS with 3% (w/v) BSA + 50 μg/ml of hscFv and left for 1 h at 37°C. Cells were washed and incubated with anti-6X His tag polyclonal antibody (OriGene) in blocking solution for 1 h at 37°C and then, with goat anti-mouse IgG1 Alexa Fluor 488 (Molecular Probes). After washing, the samples were fixed with paraformaldehyde 4% in PBS for 1 h at 4°C. After washing again, each sample was resuspended in PBS and divided into two aliquots one of which was used for immunofluorescence microscopy. The second aliquot was analysed by flow cytometer (FACScanto II, BDBioscences, Erembodegem, Belgium) equipped with three lasers (488 nm, 633 nm, 405 nm) to quantify the fluorescence intensity due to the conjugation to the hscFv compared to the cells’ autofluorescence. For the negative controls, the dialysis buffer was used in place of the hscFv, while PBS replaced the other antibodies in the controls.

### Minimum inhibitory concentration (MIC)

The hscFv antifungal activity was assessed alone and in combination with caspofungin (CAS) (Sigma-Aldrich) and amphotericin B (AMB) (Sigma-Aldrich) against *C*. *auris*. The microdilution method from EUCAST guidelines was adopted. In brief, from an overnight inoculum of *C*. *auris* in RPMI+MOPS (0.165 M pH 7), 1–5x10^5^ CFU/ml were plated in a 96-well plate. The antifungal drugs concentrations were 10-fold serially diluted starting from 4 μg/ml, while the concentrations of hscFv tested were 0.25, 2.5 and 25 μg/ml. The plates were incubated at 37°C for 24 and 48 h. The absorbance was read at 405 nm with a Microplate Reader. As reported in the guidelines, for caspofungin we considered the concentrations that inhibit 50% of yeast growth compared to drug-free control (MIC50) while for amphotericin B, the concentrations that inhibit 90% (MIC90) and 50% (MIC50) of yeast growth compared to the drug-free control. The dialysis buffer was used as negative control. The assays were performed three times in triplicate.

## Results

### Humanization of VH and VL regions of the murine monoclonal antibody 2G8

**Fig 1:**
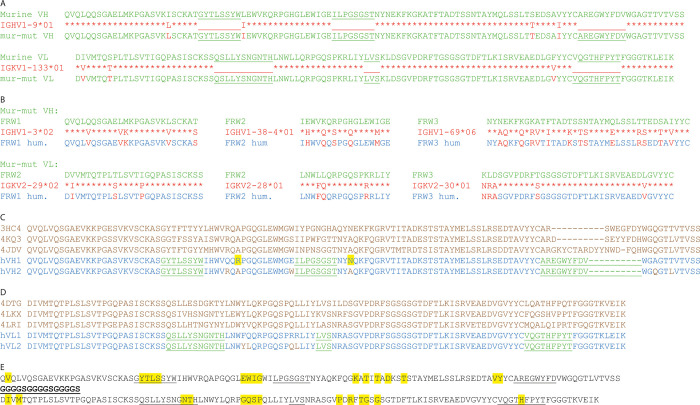
Steps of the humanization of the variable regions of the murine mAb 2G8 and scFv construction. A–first strategy: Substitution of non-homologs amino acids in VH and VL sequences; B–second strategy: Substitution of non-homologs amino acids in VH and VL frameworks; C–back mutations and mutations in the frameworks of the VH of the hscFv obtained from the second strategy; D–back mutations and mutations in the frameworks of the VL of the hscFv obtained from the second strategy; E–back-mutations in the Vernier Zones.

The murine amino acids sequence of the scFv 2G8 (VH-Linker-VL) was analyzed with ExPasy and the value of the *instability index* was 41.64. To decrease this value, the VH and VL regions were investigated separately through *“IgBlastTool”*. The murine germlines found to have the highest homology were IGHV1-9*01 for the VH (96,2%) and IGKV1-133*01 for the VL (97,7%). The substitution of the non-homologous amino acids (in VH: FRW1 I20→L, FRW2 L1→I, FRW3 S30→T, V35→I; in VL: FRW1 I2→V, S7→T, FRW3 F33→V) (Fig 1A), lowered the *instability index* to 37.05. These mutated variable regions were used for the humanization process. The first strategy showed that IGHV1-46*02 (74.7%) and IGKV2-30*01 (84.0%) were the human germlines with the highest homology. Regarding the second strategy, the human germlines found for the VH murine frameworks were: IGHV1-3*02 for FWR1 (87.5%), IGHV1-38-4*01 for FRW2 (77.1%), IGHV169*06 for FRW3 (76.3%) while for the VL murine frameworks: IGKV2-29*02 for FRW1 (84.6%), IGKV2-28*01 for FRW2 (90.7%), IGKV2-30*01 for FRW3 (86.3%) (Fig 1B). Once the frameworks had been humanized, the two single chains were assembled and launched again in ExPasy. The best *instability index*, even if still too high, was obtained through the second strategy with a value of 41.10. The VH and VL of the selected hscFv were compared with the murine ones and back-mutations (VH FRW2 S40→R and FRW3 A61→N) were carried out to decrease the *instability index* to 37.01 (Figs 1C hVH1 and 1D hVL1). Through their analysis in PDB, the sequences obtained were: 3HC4 (Identities:80%, X-RAY diffraction: 1.62 Å [33]), 4KQ3 (Identities 80%, X-RAY diffraction: 1.92 Å [34]), 4JDV (Identities 72%, X-RAY diffraction: 1.65 Å [35]) for VH and 4DTG (Identities:84%, X-RAY diffraction: 1.80 Å [36]), 4LKX (Identities 82%, X-RAY diffraction: 1.92 Å [37], 4LRI (Identities 78%, X-RAY diffraction: 1.65 Å [38]) for VL. After the analysis of amino acid sequences, non-homologous amino acids in the frameworks were changed (Fig 1C hVH2 and 1D hVL2). According to several studies [30, 39] about the critical role of Vernier Zones in protein shaping, it was found that some amino acids were back-mutated at these residues. For this reason, we did the same. The variable regions obtained were used to build the hscFv (Fig 1E).

### VL-linker-VH orientation ameliorates hscFv solubility, but it is insufficient in significantly increasing the amount of soluble protein

The recombinant hscFv cloned into the pET22b(+) vector with a VH-linker-VL orientation (VH-linker-VL-His, S1 Fig) was successfully expressed in *E*. *coli* BL21(DE3) but it was mainly found in inclusion bodies at the expected molecular weight of about 27 kDa (S2 Fig). Despite the majority of scFvs being built as VH-linker-VL, some studies have shown that the VL-linker-VH orientation ameliorates both the stability and the expression (34–37). Based on this evidence, a new expression strategy was designed to express the hscFv with this orientation (VL-linker-VH-His, S1 Fig). Unfortunately, although this approach slightly improved hscFv solubility, most of the expressed protein still precipitated in the insoluble fraction. For this reason, a preliminary purification from inclusion bodies was attempted. The protein was refolded on column and kept in 0.5 M urea; however, after isolation and concentration, it precipitated even with the addition of a surfactant (Tween 80) in the solution.

### The expression of hscFv as fusion protein with Ub increases the amount of soluble protein but impairs His tag binding to IMAC

To improve the solubility of the hscFv, the protein was expressed as a fusion protein with Ub monomer or polymers consisting of two and three head-to-tail Ub units as fusion partners (His-Ub_1_-hscFv, His-Ub_2_-hscFv and His-Ub_3_-hscFv, S1 Fig). As shown in S3 Fig, SDS-PAGE analysis and Commassie staining clearly demonstrated that the amount of the recombinant product significantly increased in the soluble faction of bacterial lysates as the number of attached ubiquitin units increases. It should be noted that while His-hscFv and His-Ub_1_-hscFv proteins showed an electrophoretic mobility which is in accordance with their expected molecular weight (~27 kDa and ~35 kDa respectively) (S3A and S3B Fig), the His-Ub_2_-hscFv and His-Ub_3_-hscFv fusion products migrated at a molecular weight lower than expected (~44 kDa and ~52 kDa respectively) (S3C and S3D Fig). Based on these results, His-Ub_2_-hscFv and His-Ub_3_-hscFv recombinant proteins were selected to be purified from the soluble fraction by IMAC. SDS-PAGE analysis of the purification process showed that the protein did not bind to the column, being completely recovered from the flow-through (S4A and S4B Fig). To evaluate if the His tag could be insufficiently exposed when the protein is in native conditions due to steric hindrance, the His-Ub_3_-hscFv protein was extracted from inclusion bodies and affinity-purified under denaturing conditions. In this case, part of the recombinant antibody was indeed captured on Ni^2+^ Sepharose matrix as demonstrated by SDS-PAGE analysis (S5 Fig). Considering these results, we hypothesized that the N-terminal His tag might be potentially masked, and for this reason, we decided to move the His tag from the N-terminus to the C-terminus.

### His tagging at the C-terminus and optimization of the purification protocol allow efficient capture of Ub_2_-hscFv on the IMAC matrix

**Fig 2:**
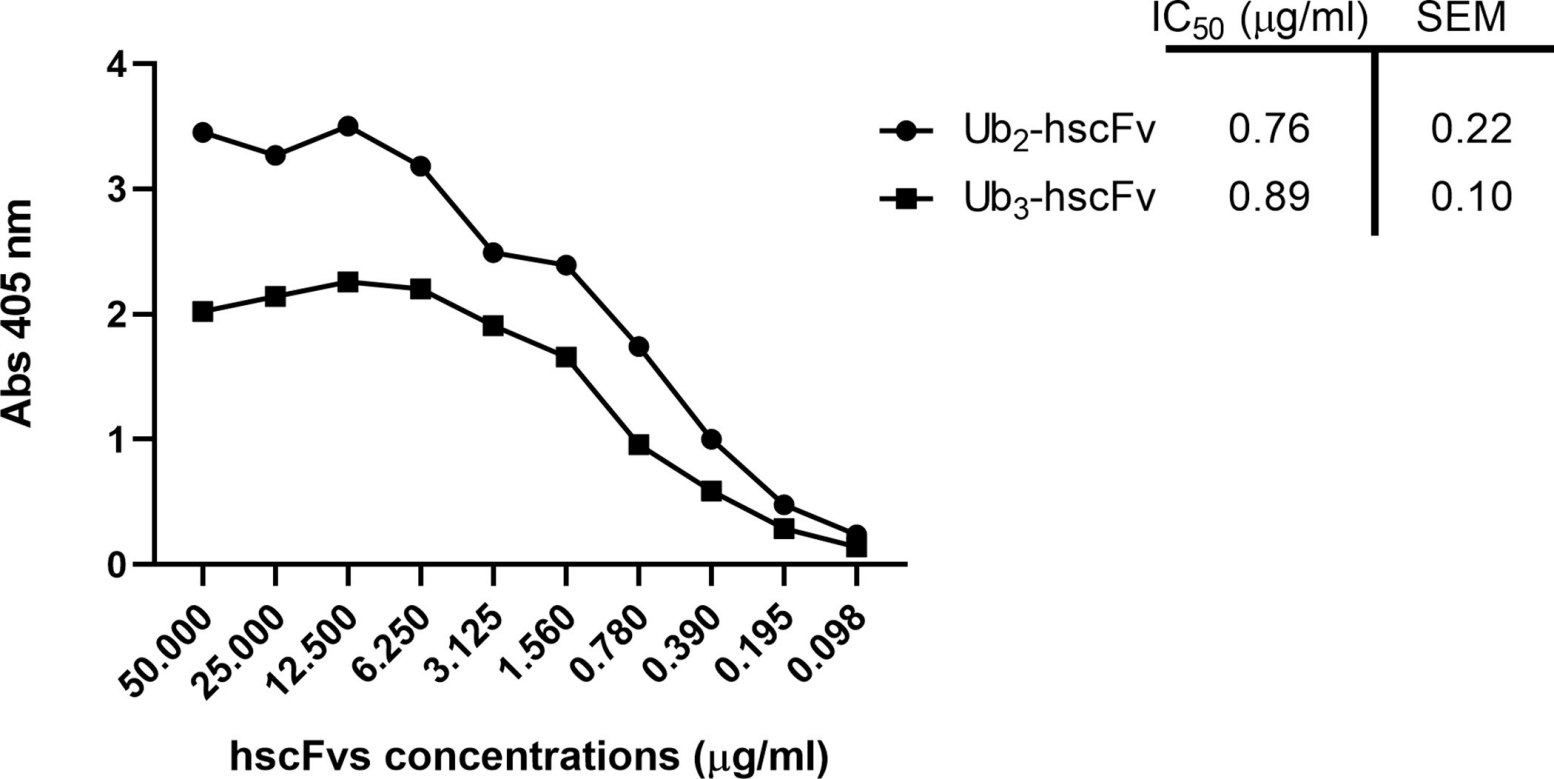
ELISA assay. Comparison of the binding activity of Ub_2_-hscFv-His and Ub_3_-hscFv-His to laminarin antigen. The IC_50_ value was calculated through GraphPad Prism 8 software using a nonlinear regression and a dose-response–inhibition equation. Data are reported as IC_50_± SEM.

To produce the Ub_2_-hscFv and Ub_3_-hscFv with a His tag at the C-terminus (Ub_2_-hscFv-His and Ub_3_-hscFv-His. S1 Fig), the coding cassettes were amplified from pET45b(+) and cloned into pET22b(+) without pel B sequence (as for pET45b(+) vector). The expression pattern followed essentially the trend seen for the N-terminal His tagged counterparts (S6A and S6B Fig). Results of the purification step revealed that the new His tag position improved protein binding to the column even though part of the protein could be still found in the flow-through (S7A and S7B Fig). Since the fusion partner can affect protein activity, the purified products were next tested in ELISA. Ub_2_-hscFv-His performed better than Ub_3_-hscFv-His in terms of their IC_50_. IC_50_ is defined as the concentration of antibody required to bind 50% of the immobilized antigen. Ub_2_-hscFv-His IC_50_ was 0.76 μg/ml while Ub_3_-hscFv-His IC_50_ was 0.89 μg/ml meaning a higher binding affinity of the former compared to the latter (Fig 2). From these results we decided to optimize the purification process and perform a preliminary characterization of only the Ub_2_-hscFv-His protein that, for simplicity, will be called just hscFv in the following paragraphs.

**Fig 3:**
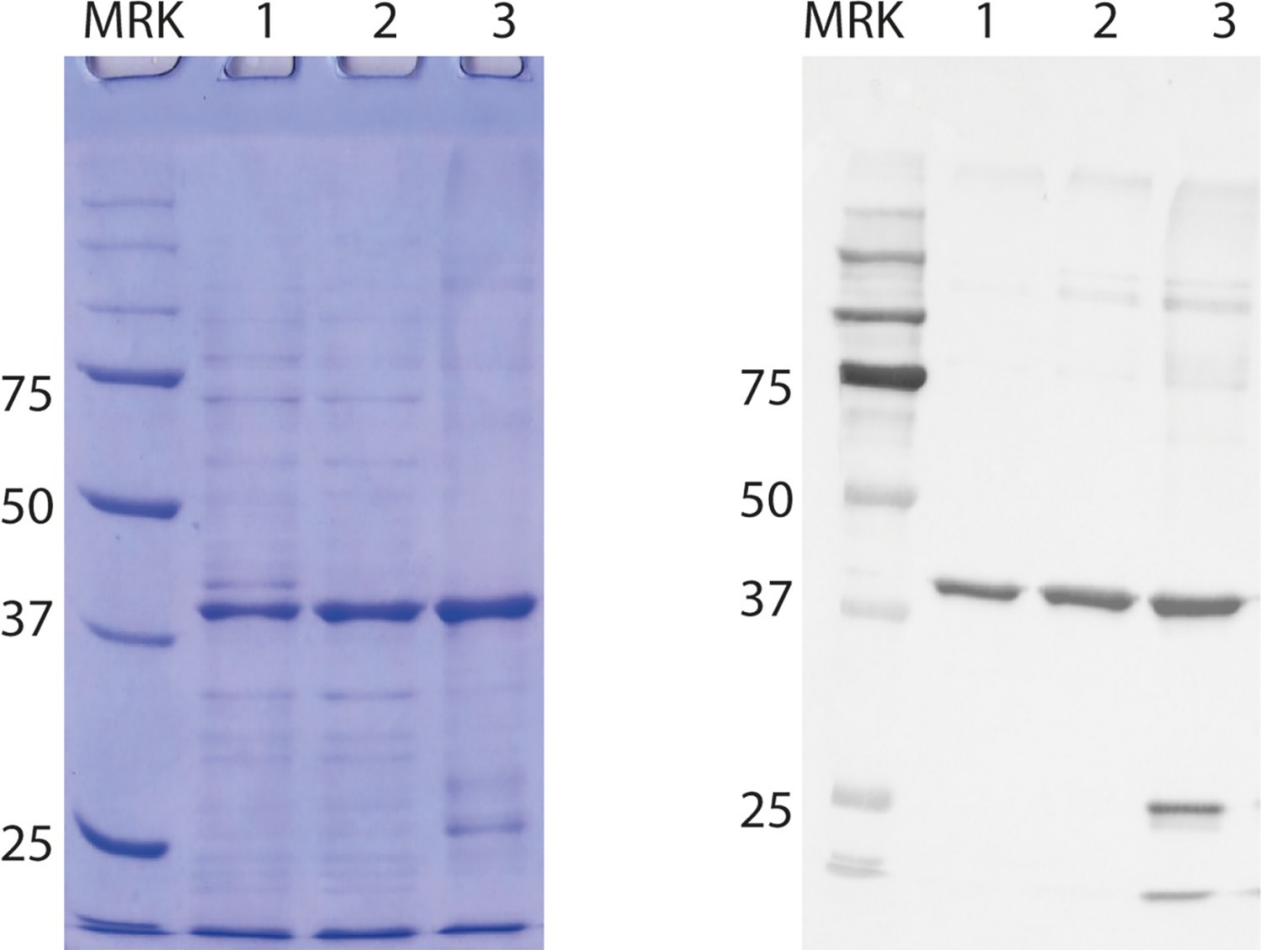
Contribution of each step of purification. Sequence of the steps of the purification process and progressive higher level of purity of the hscFv. MRK: Protein marker (kDa); 1: Starting material–soluble fraction of the bacterial cell lysate; 2: Product of the negative passage in Q Sepharose; 3: Final result of the double step (Q Sepharose + IMAC) purification.

The purification process underwent sequential optimization steps: i) changing the pH from 7.4 to 8.5 (one pH unit above the isoelectric point of the hscFv) to improve binding; ii) introducing a 100 mM imidazole wash followed by a linear gradient elution in Ni^2+^ Sepharose HP column to improve purity (S8A and S8C Fig) and, iii) a negative passage on Q Sepharose FF before IMAC with the aim to remove as many contaminants as possible (S8B Fig). As shown in S8A Fig, the protein was mainly retained by the column while most of the contaminants were removed during the washes, including the 100 mM wash. The protein eluted at around 165 mM imidazole as a single band, denoting a good level of purity. From 1L of bacterial culture, the yield was ~ 40 mg. The introduction of the first passage on the Q column resulted in the removal of a large number of contaminants with the majority of the hscFv passing in the flow-through fractions (S8B Fig) and in a cleaner recombinant product obtained after IMAC fractionation (S8C Fig). The contribution of each purification step is summarized in Fig 3 where the first lane contains the starting material (i.e. the soluble fraction of the bacterial cell lysate), the second is the protein after Q Sepharose fractionation and, the third is the protein after Q Sepharose and IMAC. The third band is cleaner from contaminants compared to the others even if it shows some products of degradation and traces of aggregates, as revealed by immunoblotting analysis. The yield obtained was 38 mg per L of bacterial culture which is very similar to the yield found with a single step of IMAC purification, highlighting that there was no significant loss of product.

### Mass spectrometry confirmed the purity of hscFv

**Fig 4:**
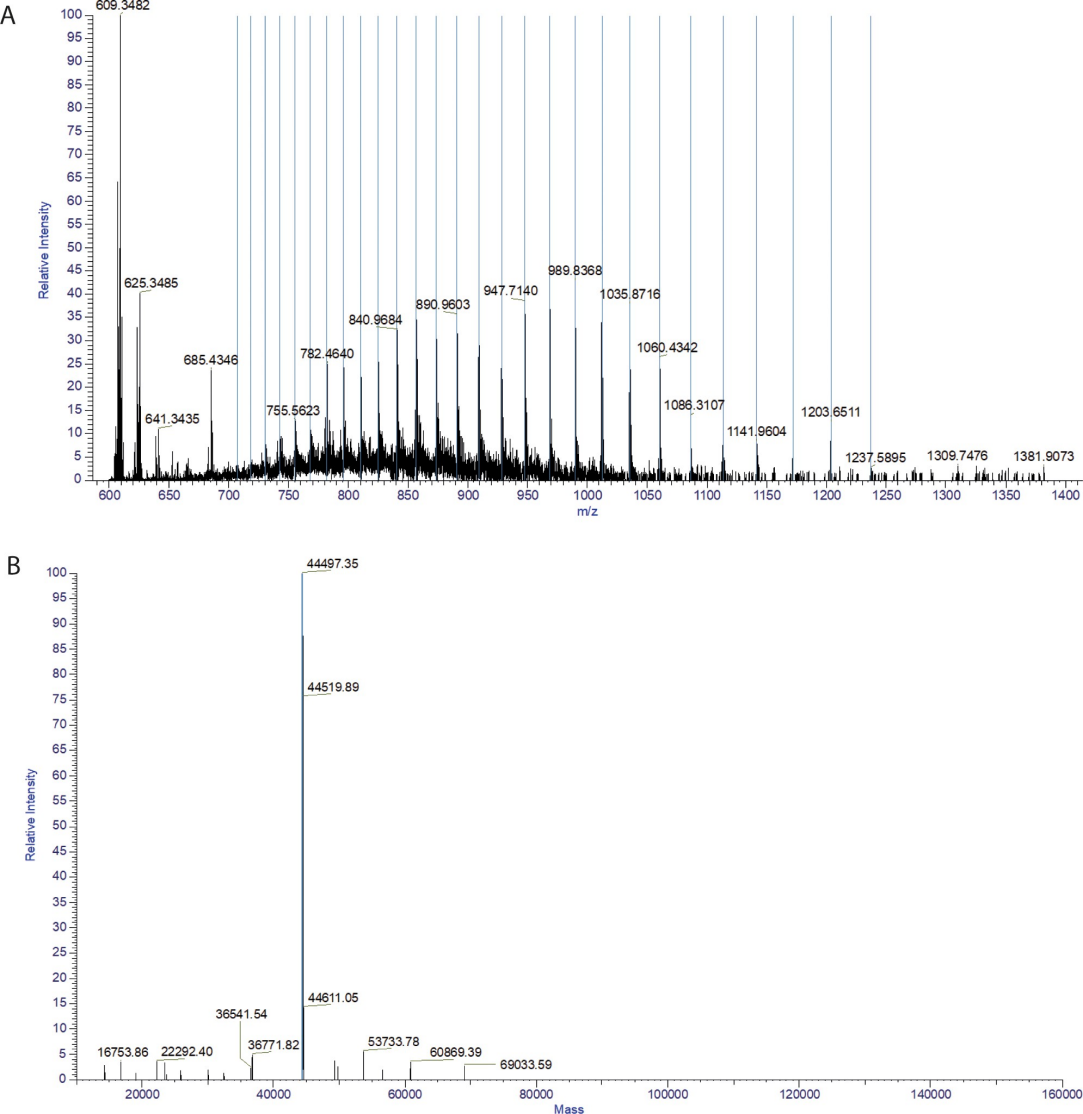
Mass spectrometry analysis. A. Positive ESI Orbitrap mass spectrum of the intact hscFv. m/z ranging from 600 to 1,400 and charge ranging from 36+ to 63+. B. Deconvoluted mass spectrum showing the prevalence of the hscFv and of its cationized adducts.

Fig 4A represents the average ESI mass spectrum with mass-to-charge (m/z) ranging between 600 and 1,400. The hscFv was analyzed in its intact form and each peak of the resulting Gaussian distribution represents a different protonated level of the proteins present in the sample. The distribution proceeds from the most protonated to the least protonated species in a charged state ranging from 36+ to 63+. The corresponding deconvoluted mass spectrum (Fig 4B) shows that the most abundant peak belongs to the hscFv and represents almost 100% of the total proteins in the sample. The molecular mass is 44,519.89 Da; the other two most abundant species are cationized adducts of the hscFv with respectively Δ mass due to 1 (44,497.35 Da) and 4 (44,611.05 Da) Na/H cation exchange. The remaining peaks are probably hscFv degradation products and residual contaminants of the purification.

### The hscFv can bind β-1,3-glucans showing an improved IC_50_

**Fig 5:**
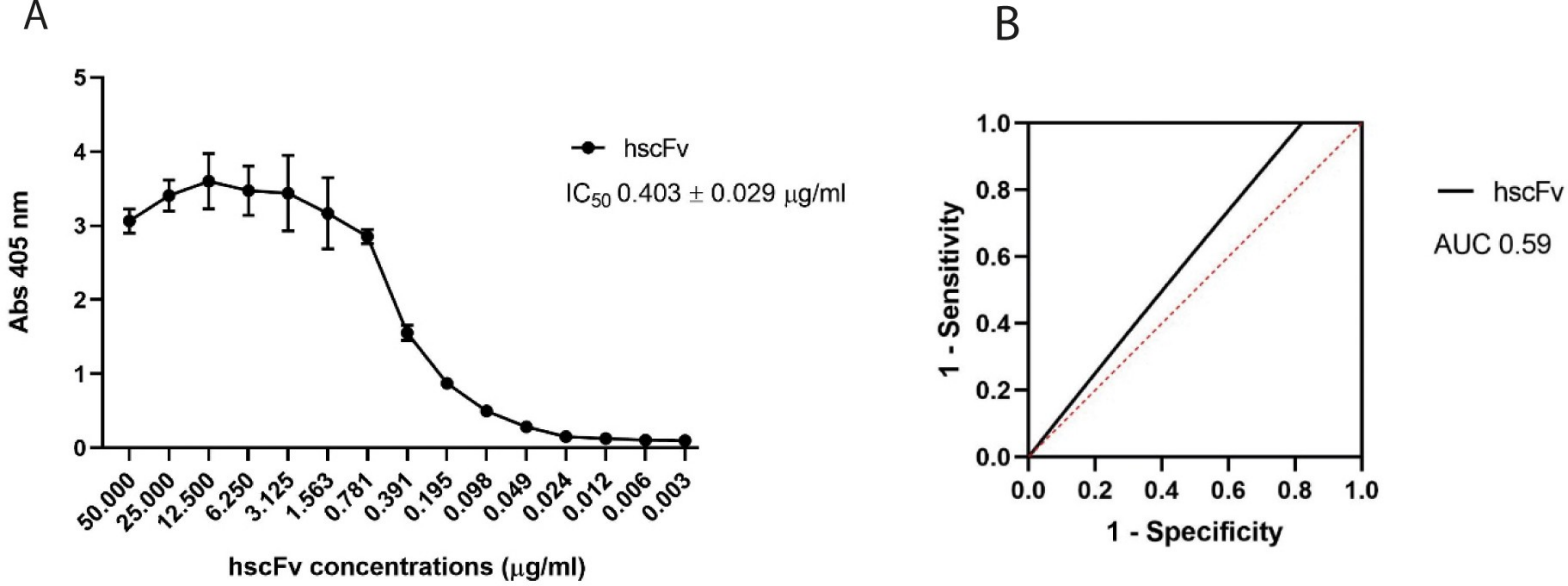
ELISA assay of the hscFv after purification with the optimized protocol. A. Evaluation of the binding of the hscFv to laminarin and calculation of the IC_50_ value. Data were plotted in GraphPad Prism 8 software and the IC_50_ was calculated with a nonlinear regression and a dose-response–inhibition equation. IC_50_ values are the mean±SEM from a triplicate. B. Evaluation of the test performance through the ROC curve. The black line is the interpolated ROC curve from which the AUC value was estimated. The red line is the random classifier threshold set at 0.5.

The hscFv derived from the optimized purification protocol was concentrated, dialyzed, and then tested in ELISA assay to assess its binding capacity to the β-1,3-glucan laminarin. The resulting IC_50_ is 0.403 ± 0.029 μg/ml (Fig 5A). This value was even lower than the IC_50_ previously calculated (Fig 2) providing further proof of the purity of the sample. From ELISA the interpolated ROC curve was extrapolated and the relative area under the curve (AUC) calculated as predictive indicators of the test performance. As shown in Fig 5B, the ROC curve of the hscFv is above the random classifier threshold and the AUC score is 0.59, which is satisfactory for specificity and sensitivity.

### The hscFv showed long-time stability and retention of binding ability when stored at 4°, -20° and, -80°C

**Fig 6:**
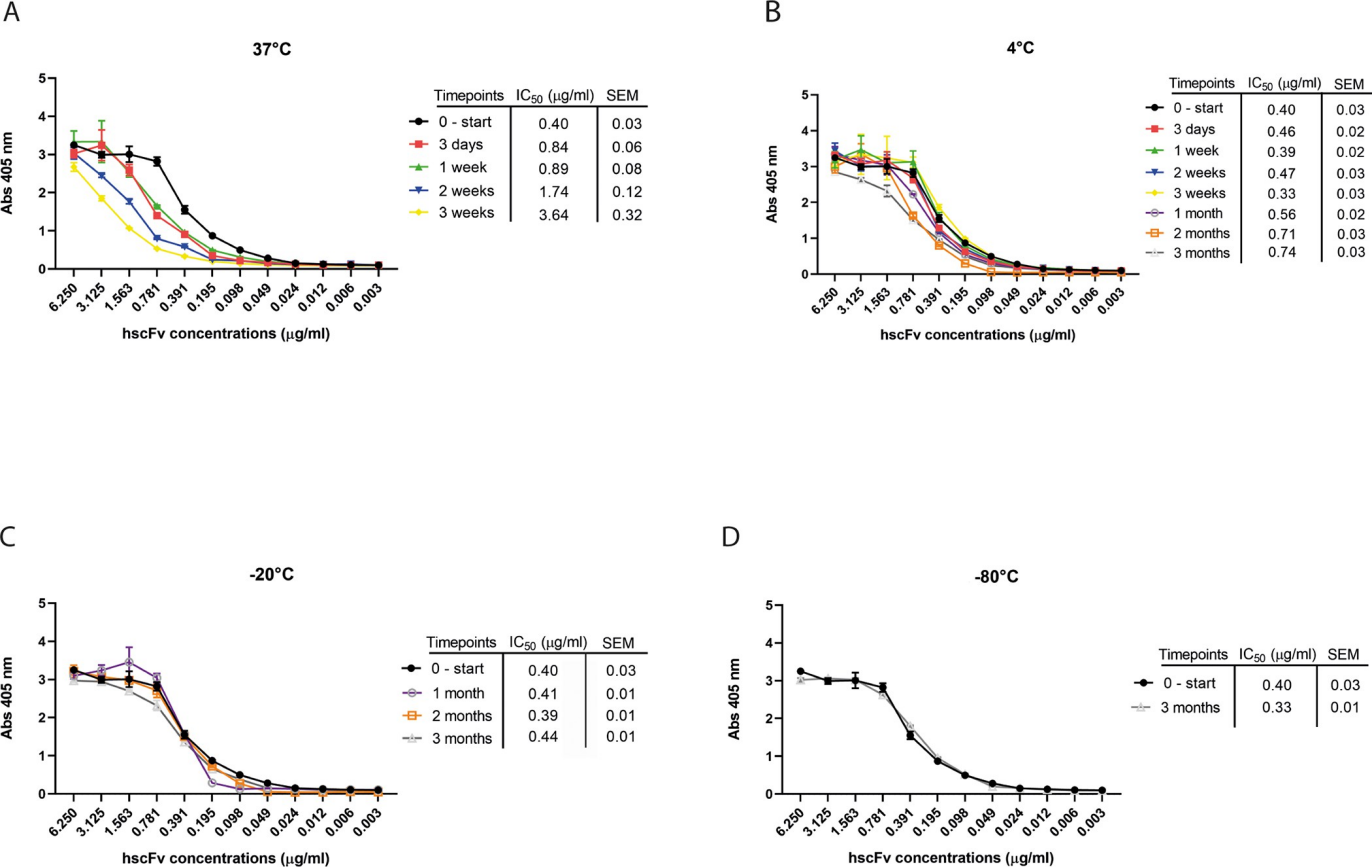
Stability test—evaluation of the binding activity of hscFv through IC_50_ calculation. hscFv was stored at different temperatures; 37°C (A), 4°C (B), -20°C (C), -80°C (D) and for different timepoints. The IC_50_ value was calculated in GraphPad Prism 8 using a nonlinear regression and a dose-response–inhibition equation. Data are the mean ± SEM from a triplicate.

Equal amounts of the same stock solution containing the purified, concentrated, and dialyzed hscFv were split into separate tubes and stored at different temperatures for different timespans. Binding ability was evaluated calculating the IC_50_ through ELISA assay, while protein stability/integrity was determined by SDS-PAGE followed by Coomassie staining and western immunoblotting. Already after 3 days at 37°C, the hscFv started losing its binding ability (Fig 6A) and stability (S9B Fig) as demonstrated by the gradual disappearance of the monomeric band in favor of aggregate formation (S9C–S9E Fig). At 4°C the binding capacity of the hscFv was preserved for several weeks, with IC_50_ values not significantly different to the initial value (Fig 6B). Only after one month the IC_50_ progressively increased and the aggregates intensify compared to the aliquots left at -20° and -80°C (S9F–S9H Fig). Moreover, the hscFv stored at -20° and -80°C retained a binding capacity similar to the values exhibited at the beginning of the stability test (Fig 6C and 6D) and remained mainly in the monomeric form, preserving it from aggregate formation, in contrast to the 4°C sample (S9F–S9H Fig).

### The hscFv can bind β-1,3-glucans of *C*. *auris* and *C*. *albicans* cell wall

**Fig 7:**
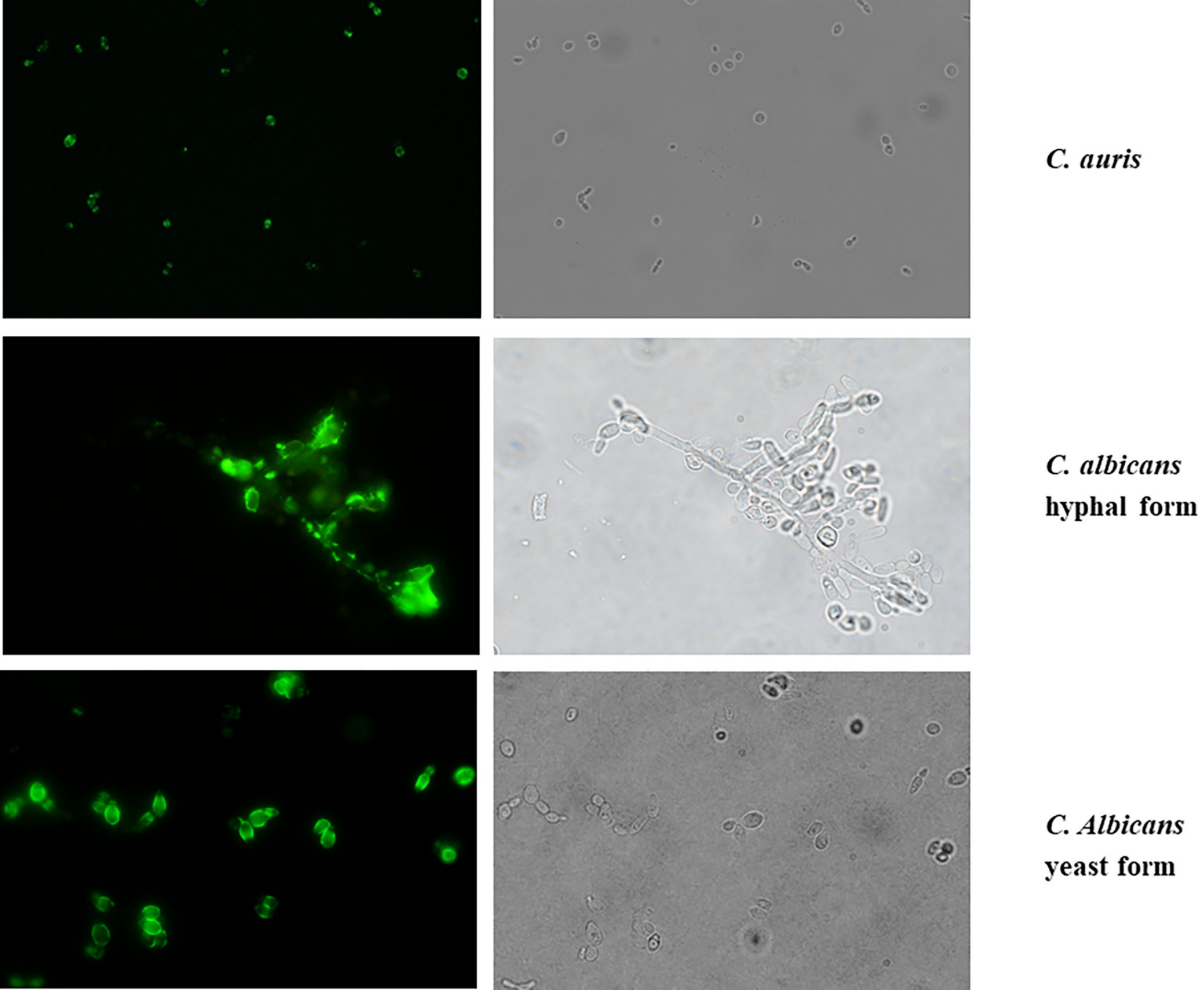
Immunofluorescence analysis of *C*. *auris* and *C*. *albicans* labelled with the hscFv. Green fluorescence and bright-field images.

Until now the binding of hscFv to β-1,3-glucans was evaluated only by ELISA assays and using laminarin as antigen. Here we demonstrated its ability in recognizing and binding β-1,3-glucans on the cell wall of *C*. *auris* and *C*. *albicans* both in hyphal and yeast form through immunofluorescence and flow cytometry (Fig 7 and S10 Fig respectively). Through flow cytometry analysis the fold-increase between the fluorescence of treated samples and the basal cell autofluorescence was calculated. For *C*. *auris* the fold-increase is ~50 while for *C*. *albicans* is ~30.5. By considering only the events at higher forward scatter (FSC) and side scatter (SSC) (blue events), these values increase considerably: for *C*. *auris* the value becomes 120 and for *C*. *albicans* 54. The subpopulations considered correspond to the budded cells for *C*. *auris* and to the hyphae for *C*. *albicans* and represent respectively the 13% and the 66% of the entire samples (S10 Fig).

### The hscFv increases the activity of caspofungin and amphotericin B against *C*. *auris*

Confident about the results obtained with the humanized full-length antibody H5K1 [24], the hscFv was also tested in combination with caspofungin (CAS) (Sigma-Aldrich) and amphotericin B (AMB) (Sigma-Aldrich) against *C*. *auris*. EUCAST guidelines for the microdilution method were followed. We considered MIC50 as the lowest concentration that inhibits 50% of the growth compared to drug-free control and MIC90 as the lowest concentration that inhibits 90% of the growth compared to drug-free control. For caspofungin, positive results were visible already at 24 hours with the gradual loss of efficacy of CAS from 0.125 μg/ml and the retention of activity when combined with hscFv (S11A Fig). In particular, the combination with 25 μg/ml of hscFv resulted in a 2-fold shift of the MIC50 dilution, from 0.125 μg/ml of caspofungin to 0.03125 μg/ml (Table 4). At 48 hours the effect remains (S11B Fig): the MIC50 2-fold shift is maintained for the combination with 25 μg/ml of hscFv (from 0.25 to 0.0625 μg/ml of caspofungin) and is 1-fold with 2.5 μg/ml of hscFv (from 0.25 to 0.125 μg/ml) (Table 4). Nevertheless, the best results were obtained with amphotericin B for which we considered both MIC90 and MIC50 (S12A and S12B Fig). At 24 hours both the combination with 2.5 μg/ml and with 25 μg/ml of hscFv caused a 1- and 2-fold shift respectively, for both MIC90 and MIC50 (MIC90 decreased from 0.25 μg/ml to 0.125 μg/ml with 2.5 μg/ml of hscFv and to 0.0625 μg/ml with 25 μg/ml of hscFv, whereas MIC50 decreased from 0.125 μg/ml to 0.0625 μg/ml when AMB is combined with 2.5 μg/ml of hscFv and to 0.03125 μg/ml when combined with 25 μg/ml of hscFv) (Table 5). The MIC50 shift is also observable using 0.25 μg/ml of hscFv at 48 hours. While the fold-shifts for the combinations with 2.5 and 25 μg/ml of hscFv remained constant for MIC90, they increased 1-fold for MIC50 (MIC90 decreased from 0.5 μg/ml to 0.25 with the addition of 2.5 μg/ml of hscFv and to 0.125 μg/ml with 25 μg/ml of hscFv while MIC50 decreased from 0.5 μg/ml to 0.25 μg/ml with 0.25 μg/ml of hscFv, to 0.125 μg/ml with 2.5 μg/ml of hscFv and to 0.0625 μg/ml with 25 μg/ml of hscFv) (Table 5). Table 6 confirmed the data obtained in the MIC experiments showing the IC_50_ values calculated from the resulting growths. IC_50_ is defined as the concentration of the agent/s required to inhibit the 50% of the fungal growth. A progressive lowering of the IC_50_ was found when CAS and AMB were used in combination with increasing concentrations of hscFv, further demonstrating the enhancing capacity of hscFv towards these drugs. When combined with CAS, 25 μg/ml of hscFv decreased the IC_50_ concentration more than 3 times at 24 hours and almost 5 times at 48 hours compared to the control. At 24 hours 2.5 μg/ml of hscFv is unable to reduce the CAS’ IC_50_ significantly however, at 48 hours it reduces the value by almost a half. With AMB, both at 24 and 48 hours, the IC_50_ is reduced by at least a half with of 2.5 μg/ml of hscFv and at least by 4 times with 25 μg/ml of hscFv.

**Table 4:** Percentage of growth inhibition ± SD.

	% *C*. *auris* growth inhibition							
Drug μg/ml	CAS	Combination CAS-hscFv (μg/ml)			CAS	Combination CAS-hscFv (μg/ml)		
		0.25	2.5	25		0.25	2.5	25
**4**	93.0±2.0	94.3±4.9	96.3±2.8	90.6±2.1	77.2±0.9	78.5±2.6	78.8±2.6	77.6±4.2
**2**	98.6±1.2	98.9±2.7	98.6±1.9	94.7±2.4	91.9±1.0	91.7±1.1	93.9±2.2	90.2±2.5
**1**	99.1±0.8	98.8±0.7	98.7±1.9	95.1±1.7	94.7±0.9	93.2±1.2	95.6±1.7	92.7±2.0
**0.5**	99.0±0.4	98.8±0.9	100±1.8	96.2±1.6	91.6±2.0	91.4±1.1	95.6±1.3	94.1±1.1
**0.25**	97.0±3.2	98.0±1.3	100±2.1	96.3±1.1	56.3±10.5*	55.4±7.8*	88.4±2.8	93.3±0.6
**0.125**	59.2±17.0*	68.2±9.9*	85.0±4.1*	95.8±1.2	6.5±8.2	10.7±4.1	53.4±7.4*	91.1±0.6
**0.0625**	9.1±13.4	24.7±19.5	29.8±4.9	91.4±1.9	0.7±3.5	2.8±4.1	5.4±6.7	71.2±10.6*
**0.03125**	0±12.2	6.7±15.3	11.9±4.3	61.5±11.5*	5.2±3.2	6.3±3.2	3.4±2.6	26.6±5.2
**0.156**	0±13.5	2.4±19.8	16.2±7.8	15.1±8.8	6.9±4.6	9.5±3.4	11.6±5.9	20.1±8.8
**0.0078**	0±16.0	2.4±15.0	12.8±5.0	9.8±9.3	3.9±3.3	6.9±3.6	7.7±2.8	12.0±3.1
	**24h**				**48h**			

*C*. *auris* cells were treated with caspofungin (CAS) alone and in combination with hscFv. Absorbance was read at 405 nm after 24 hours and 48 hours of incubation. Data are reported as mean ± SD of the percentage of inhibition of the fungal growth obtained from three experiments performed in triplicate. MIC50 breakpoints are marked with ⁎.

**Table 5:** Percentage of growth inhibition ± SD.

	% *C*. *auris* growth inhibition							
Drug μg/ml	AMB	Combination AMB-hscFv (μg/ml)			AMB	Combination AMB-hscFv (μg/ml)		
		0.25	2.5	25		0.25	2.5	25
**4**	100±2.9	100±1.5	100±2.7	100±2.9	100±0.7	100±0.7	100±1.1	100±1.1
**2**	100±2.4	100±2.1	100±2.8	100±2.6	100±1.0	100±0.9	100±1.1	100±1.0
**1**	100±3.2	100±1.3	100±2.9	100±1.9	100±1.4	100±0.5	100±1.0	100±0.6
**0.5**	100±2.0	100±1.0	100±1.2	98.9±1.9	100±1.0*†	100±0.4**†**	100±0.9	100±0.7
**0.25**	95.6±3.2**†**	99.0±1.8**†**	100±2.0	98.2±1.1	36.1±8.9	50.0±22.4*	96.0±4.6**†**	100±0.4
**0.125**	70.2±19.3*	80.1±16.0*	100±1.6**†**	98.4±1.5	18.6±4.6	15.9±6.7	50.1±10.1*	94.9±4.3**†**
**0.0625**	14.7±19.0	31.8±26.2	75.1±4.1*	91.3±7.9**†**	3.1±3.2	5.9±2.8	15.9±2.8	50.4±17.8*
**0.03125**	0±9.9	8.9±14.8	31.8±14.6	71.0±14.8*	2.3±2.9	0±2.3	6.1±4.2	29.4±8.0
**0.156**	0±11.6	9.3±16.3	22.2±6.3	47.3±18.8	1.3±2.8	4.2±2.4	6.2±6.1	18.8±8.1
**0.0078**	0±9.4	7.6±14.7	14.4±9.6	21.5±17.1	0±4.6	3.5±5.0	2.6±3.5	13.3±12.7
	**24h**				**48h**			

*C*. *auris* cells were treated with different concentrations of amphotericin B (AMB) alone and in combination with different concentrations of hscFv. Absorbance was read at 405 nm after 24 hours and 48 hours of incubation. Data are reported as mean ± SD of the percentage of inhibition of the fungal growth obtained from three experiments performed in triplicate. MIC50 breakpoints are marked with

⁎ while MIC90 breakpoints with †.

**Table 6:** IC_50_ (μg/ml) ± SEM value determined at 24 hours and 48 hours.

	24h		48h	
	IC_50_ (μg/ml)	SEM	IC_50_ (μg/ml)	SEM
CAS	0.104	0.005	0.225	0.055
CAS + 0.25 μg/ml hscFv	0.095	0.006	0.230	0.053
CAS + 2.5 μg/ml hscFv	0.086	0.002	0.104	0.025
CAS + 25 μg/ml hscFv	0.028	0.001	0.022	0.006
AMB	0.102	0.005	0.288	0.008
AMB + 0.25 μg/ml hscFv	0.089	0.006	0.254	0.016
AMB + 2.5 μg/ml hscFv	0.050	0.002	0.133	0.004
AMB + 25 μg/ml hscFv	0.025	0.006	0.064	0.003

*C*. *auris* cells were treated with caspofungin (CAS) or amphotericin B (AMB) alone or in combination with different concentrations of hscFv. The IC_50_ value of CAS and AMB was calculated from a dose-response–inhibition equation in nonlinear regression using the GraphPad Prism 8 software. Data are reported as mean ± SEM of the values obtained from three experiments performed in triplicate.

## Discussion

Considering the increasing interest of pharmaceutical companies and research laboratories in antibody-based treatments, the development of a humanized scFv is an intriguing challenge [6, 40, 41]. The IgG2b antibody 2G8 was an optimal candidate for this kind of study as it not only binds β-1,3-glucans (which are fundamental components of the fungal cell wall) but also demonstrated its efficacy in the inhibition of growth, adhesion and infection progression, against several pathogenic fungi both *in vitro* and *in vivo* [15–17]. The first result of a humanization of 2G8 was the full-length antibody H5K1 [24] but our manuscript shows for the first time the use of 2G8 for the development of a humanized antibody fragment. Herein we report the development and the initial characterization of Ub_2_-hscFv-His (hscFv), which is the result of a humanization process followed by a detailed study aimed to express in *E*. *coli* and purify a considerable amount of clean product.

The production of engineered proteins in bacteria has several advantages, including the possibility to obtain large amounts of product at low cost and the ability to easily scale-up the system. However, formation of aggregates in large-scale bacterial scFv expressions [40, 42] is clearly problematic especially in terms of therapeutic applicability. Therefore, solubility is crucial for the development of an efficient scFv. The VL-VH orientation was preferred because it provided a slightly more soluble product than the VH-VL, but the real contribution in terms of solubility was given by the introduction of ubiquitin as a fusion partner at the N-terminus. Ubiquitin (Ub) is a small, stable and highly conserved protein expressed in all eukaryotic cells. It has long been used as a tag to enhance fusion expression by increasing the solubility and stability of the expressed partner peptides and protecting them from proteolytic degradation in prokaryotic hosts [20, 23, 43–46]. In our case, for hscFv expression, the solubility increased with the number of ubiquitin monomers added to the sequence, but at the same time, the steric hindrance made the His tag-based purification difficult for both the His-Ub_2_- and His-Ub_3_-hscFv. The transfer of the His tag at the C-terminus consistently improved the purification profile although the hscFvs were still largely found in the flow-through. We believed that protein folding could still mask the C-terminal His tag interfering with the binding to the column. The pH modification from the standard 7.4 to 8.5 led to total protein binding, thus suggesting that a temporary and reversible conformational change occurred under basic conditions resulting in a more favorable exposure of the His tag. Moreover, the addition of a negative passage in Q Sepharose FF before IMAC, and of a 100 mM imidazole wash in IMAC, further improved the fraction purity without excessively affecting the overall yield of ~38 mg. The mass spectra confirmed the high purity of the collected fractions since almost 100% of the total proteins in the sample was represented by the hscFv which has a molecular mass of 44,519.89 Da, and by its cationized adducts. Finally, the hscFv was able to recognize and bind β-1,3-glucans in ELISA with an IC_50_ of 0.403 ± 0.029 μg/ml. The lower value of IC_50_ compared to the evaluation performed before the optimized purification protocol is a further demonstration of the improved purity of the hscFv. Indeed, it has been demonstrated that contaminants can negatively affect the binding in ELISA tests [47].

Extracted proteins may not remain soluble and therefore active when stored for extended period of time hence, it is important to find the optimal storage conditions and monitor the structural integrity and the binding efficiency overtime. Due to these limits and complications that often affect scFv development, our hscFv was analysed to assess its solubility and activity after storage at different temperatures and for different timepoints. The observation of the binding whilst also monitoring aggregate formation comes from evidence in the literature of scFvs that maintain their binding capacity and activity even in presence of small oligomers or soluble aggregates [48]. In our case the formation of many aggregates and the total loss of the binding occurred within 3 weeks at 37°C while at 4°C, the hscFv maintained the binding capacity for longer with a slight decrease only after one month. At 4°C aggregates also increased, especially when compared to the samples stored at -20° and -80°C which, on the contrary, better preserved both the binding ability and the monomeric form.

Nowadays, the rapid diffusion of resistance to the commercially available antifungal drugs among pathogenic fungi is threatening the public health. In addition, new species intrinsically and multidrug resistant like *C*. *auris* are rapidly spreading worldwide [49, 50]. *Cryptococcus*, *Aspergillus* and *Candida* spp. are responsible every year for the majority of fungal infections and the highest mortality rate [51, 52], but the latter has become the most frequent type of hospital acquired infection especially in patients with critical or chronic illness [53]. New strategies to fight the fungal burden are urgently needed and the use of biological compounds is an exquisite approach. In the past few years Mycograb, a scFv targeting HSP90, reached clinical trials but never achieved marketing authorization especially for solubility issues and aggregate formation [14, 54]. Given the positive results of our hscFv in terms of stability and activity, we evaluated the binding also on *C*. *auris* and *C*. *albicans* cells, the latter both in hyphal and yeast form. The fold-increase of the mean fluorescence intensity in *C*. *auris* and *C*. *albicans* were respectively 50 and 30.5, but they considerably increased to 120 and 54 considering the subpopulations composed by budded cells for *C*. *auris* and cells in hyphal form for *C*. *albicans*. These subpopulations represented respectively 13% and 66% of the entire samples and were also the cells with the highest positivity for the hscFv. This was consistent with the information reported in the literature showing a higher β-1,3-glucan exposition in bud scars and in hyphae [55, 56]. Finally, considering the great concern generated by *Candida auris* for its intrinsic lower susceptibility to commercially available antifungal drugs [50, 57, 58], we were interested in evaluating the effectiveness of the hscFv in affecting its growth, hence we used it as fungal model. Its activity against *C*. *auris* was assessed through MIC assays in combination with caspofungin and amphotericin B. The hscFv alone did not affect the fungal growth but it contributed substantially to the activity of both caspofungin and amphotericin B especially at concentrations at which they are less efficacious. MIC50 of caspofungin shifted 2-fold with 25 μg/ml of hscFv at 24 and 48 hours and 1-fold with 2.5 μg/ml at 48 hours. With amphotericin B, the hscFv reached its greatest potential with a decrease of MIC50 and MIC90 concentration by 1-fold with 2.5 μg/ml of hscFv, and by 2 folds with 25 μg/ml at 24 hours. At 48 hours, the MIC90 shift trend remained the same with 2.5 and 25 μg/ml of hscFv while, MIC50 concentration shifted 1-fold with 0.25 μg/ml of hscFv, by 2 fold with 2.5 μg/ml and by 3 fold with 25 μg/ml. The enhancing effect of the hscFv toward caspofungin and amphotericin B obtained in the MIC assays was consistent with the data obtained calculating the IC_50_. 25 μg/ml of hscFv was able to reduce the IC_50_ by more than 3 times at 24 hours, almost 5 times at 48 hours when combined with CAS, and by at least 4 times both at 24 and 48 hours with AMB. On the other hand, 2.5 μg/ml of hscFv combined with CAS decreased the IC_50_ almost by a half compared to the drug alone at 48 hours. When combined with AMB it lowered it at least by a half both at 24 and at 48 hours. The antifungal activity of the hscFv used in combination with caspofungin and amphotericin B was strongly comparable with that of the humanized full-length H5K1 with which it shares the same murine counterpart [24]. Moreover, the effective concentrations used in this work were even lower and this finding adds even more value to our product since formulations with high protein concentrations often led to heavy aggregate formation when tested *in vivo*. Additionally, the rationale behind the choice to produce a scFv comes from the idea to develop an hscFv-targeted delivery system carrying an antifungal drug able to exert additive or synergic effects with the hscFv. This approach should allow to reduce the dosage and the posology of the antifungal drugs currently employed in clinics, thus limiting their severe side effects.

## Conclusion

The use of biological drugs in the fight against pathogenic fungi has been hindered by several obstacles and limited research. In this work we have developed a new expression strategy to improve hscFv solubility and purity involving VH-VL orientation, the use of head-to-tail Ub polymers as fusion partners, the correct positioning of the His tag and optimization of the purification scheme with the introduction of an ion exchange chromatographic step of fractionation.

The produced hscFv proved to be efficient in binding β-1,3-glucans and in labelling *C*. *auris* and *C*. *albicans* cells, showing also good stability when stored at temperatures ≤4°C.

The hscFv was able to increase the potencies of commonly used antifungal drugs, thus being useful in the treatment of fungal infections as co-drug.

Further experiments will be directed to evaluate the effects of our hscFv in combination with other antifungals and against other fungal species, including resistant strains. Given the small size of the hscFv, it may be interesting to determine binding, activity and penetration within biofilms, as well as its possible use in developing targeted delivery systems.

## Supporting information

S1 Fig Vectors and constructs used in this study. Summarizing list of all the vectors used (A and D) and constructs elaborated and produced in sequence. The VH-linker-VL and VL-linker-VH constructs in pET22b(+) vector were created to assess the best orientation (B and C). The VL-linker-VH orientation was the most soluble, hence it was chosen for the next studies and was denominated hscFv. The coding sequence for one or more ubiquitin (Ub) monomers was inserted in pET45b(+) (E, F and G) and then the hscFv was subcloned in pET45b(+) vector empty or already cloned with Ub monomer/s (H, I, J and K). His-Ub_2_-hscFv and His-Ub_3_-hscFv proteins could not be purified either from soluble fraction or from inclusion bodies under denaturing conditions. This was probably due to the ubiquitin hindrance that impairs the binding to the column during affinity chromatography. Therefore, the constructs Ub_2_-hscFv and Ub_3_-hscFv were cloned in pET22b(+) producing the Ub_2_-hscFv-His and Ub_3_-hscFv-His that were properly purified and tested in ELISA (L and M). Ub_2_-hscFv-His performed better and was used for the *in vitro* tests.
(PDF)

S2 Fig SDS-PAGE of hscFvs’ expressions. A. VH-linker-VL orientation, B. VL-linker-VH orientation. MRK: protein markers (kDa); NI: not induced; 1/2/3h: hours after induction; Soluble: soluble fractions of protein; IBs: inclusion bodies, hence insoluble fractions of proteins. Arrows indicate the position of the hscFv recombinant proteins.
(PDF)

S3 Fig SDS-PAGE expression analysis of hscFv proteins in pET45b(+). A. His-hscFv; B. His-Ub_1_-hscFv; C. His-Ub_2_-hscFv; D. His-Ub_3_-hscFv. Arrows indicate the position of the hscFv recombinant proteins.
(PDF)

S4 Fig Purification of Ub fusion hscFv proteins from the soluble fraction. A. His-Ub_2_-hscFv; B. His-Ub_3_-hscFv. MRK:protein marker (kDa); NI: not induced; C. sample loaded into the column; FT: flow-through. Arrows indicate the position of the hscFv recombinant proteins.
(PDF)

S5 Fig Purification of His-Ub_3_-hscFv from inclusion bodies. MRK: protein marker (kDa); NI: not induced; C: sample loaded into the column; FT: flow-through. Arrow indicates the position of the hscFv recombinant protein.
(PDF)

S6 Fig SDS-PAGE analysis of Ub_2_-hscFv-His (A) and Ub_3_-hscFv-His (B). MRK: protein marker (kDa); NI: not induced; 1/2/3h: hours after induction; Soluble: soluble fractions of protein; IBs: inclusion bodies, hence insoluble fractions of proteins. Arrows indicate the position of the hscFv recombinant protein.
(PDF)

S7 Fig Purification of Ub_2_-hscFv-His (A) and Ub_3_-hscFv-His (B) from the soluble fraction. MRK: protein marker (kDa); C: sample loaded into the column; FT: flow-through.
(PDF)

S8 Fig Passages of the optimized purification protocol. A. Single passage purification of Ub_2_-hscFv-His at pH 8.5, step at 100 mM of imidazole and gradient to 250 mM of imidazole. B-C. Double passage purification of Ub_2_-hscFv-His: negative passage in Q Sepharose FF at pH 7.4, elution with NaCl (B) and positive passage in IMAC at pH 8.5, step at 100 mM of imidazole and gradient to 250 mM of imidazole (C). MRK: protein marker (kDa); C: sample loaded onto the column; FT: flow-through.
(PDF)

S9 Fig Stability test—evaluation of the formation of aggregates after storing the hscFv at different temperatures and for different timepoints. hscFv was stored at different temperatures; 37°C, 4°C, -20°C, -80°C, and for different timepoints. The solubility and the integrity of the recombinant protein was evaluated through SDS-PAGE and western immunoblotting. The samples were loaded in 10% polyacrylamide gels and stained with Brilliant Blue Coomassie R-250 or electroblotted onto a nitrocellulose membrane for protein detection.
(PDF)

S10 Fig Flow cytometric analysis of MFI of *C*. *auris* and *C*. *albicans* cells treated with hscFv. From a FSC vs SSC contour plot (depicting cell physical characteristics) specific gates surround green and blue events. On the left *C*. *auris* cells: in red the entire sample population; in green the subpopulation of the unbudded cells; in blue the subpopulation of the budded cells. On the right *C*. *albicans* cells: in red the entire sample population; in green the subpopulation of the cells in yeast form; in blue the subpopulation of the cells in hyphal form.
(PDF)

S11 Fig MIC assay of caspofungin (CAS) alone and in combination with hscFv. Caspofungin was tested alone and in combination with different concentrations of hscFv against *C*. *auris*. The histograms represent the fungal growth as determined by measuring the Abs at 405 nm. The mean ± SD of the Abs read after 24 (A) and 48 (B) hours were obtained from three independent experiments performed in triplicate. The growth of untreated cells was used as control. With MIC50 we considered the lowest concentrations that inhibit 50% of the fungal growth compared to the drug-free control (the red line threshold).
(PDF)

S12 Fig MIC assay of amphotericin B (AMB) alone and in combination with hscFv. Amphotericin B was tested alone and in combination with different concentrations of hscFv against *C*. *auris*. The histograms represent the fungal growth as determined by measuring the Abs at 405 nm. The mean ± SD of the Abs read after 24 (A) and 48 (B) hours were obtained from three independent experiments performed in triplicate. The growth of untreated cells was used as control. With MIC50 we considered the lowest concentrations that inhibit 50% of the fungal growth compared to the drug-free control (the red line threshold) while with MIC90 the lowest concentrations that inhibit 90% of the fungal growth compared to the drug-free control (the green line threshold).
(PDF)

S1 Raw images (PDF)
