# Supplementary material for: Development and in vitro characterization of a humanized scFv against fungal infections
Source: PLoS One. 2022 Oct 31;17(10):e0276786. doi: 10.1371/journal.pone.0276786 (PMC9621433; doi:10.1371/journal.pone.0276786)
Supplement: S1 Fig — Summarizing list of all the vectors used (A and D) and constructs elaborated and produced in sequence. The VH-linker-VL and VL-linker-VH constructs in pET22b(+) vector were created to assess the best orientation (B and C). The VL-linker-VH orientation was the most soluble, hence it was chosen for the next studies and was denominated hscFv. The coding sequence for one or more ubiquitin (Ub) monomers was inserted in pET45b(+) (E, F and G) and then the hscFv was subcloned in pET45b(+) vector empty or already cloned with Ub monomer/s (H, I, J and K). His-Ub2-hscFv and His-Ub3-hscFv proteins could not be purified either from soluble fraction or from inclusion bodies under denaturing conditions. This was probably due to the ubiquitin hindrance that impairs the binding to the column during affinity chromatography. Therefore, the constructs Ub2-hscFv and Ub3-hscFv were cloned in pET22b(+) producing the Ub2-hscFv-His and Ub3-hscFv-His that were properly purified and tested in ELISA (L and M). Ub2-hscFv-His performed better and was used for the in vitro tests. (PDF) [file pone.0276786.s001.pdf]

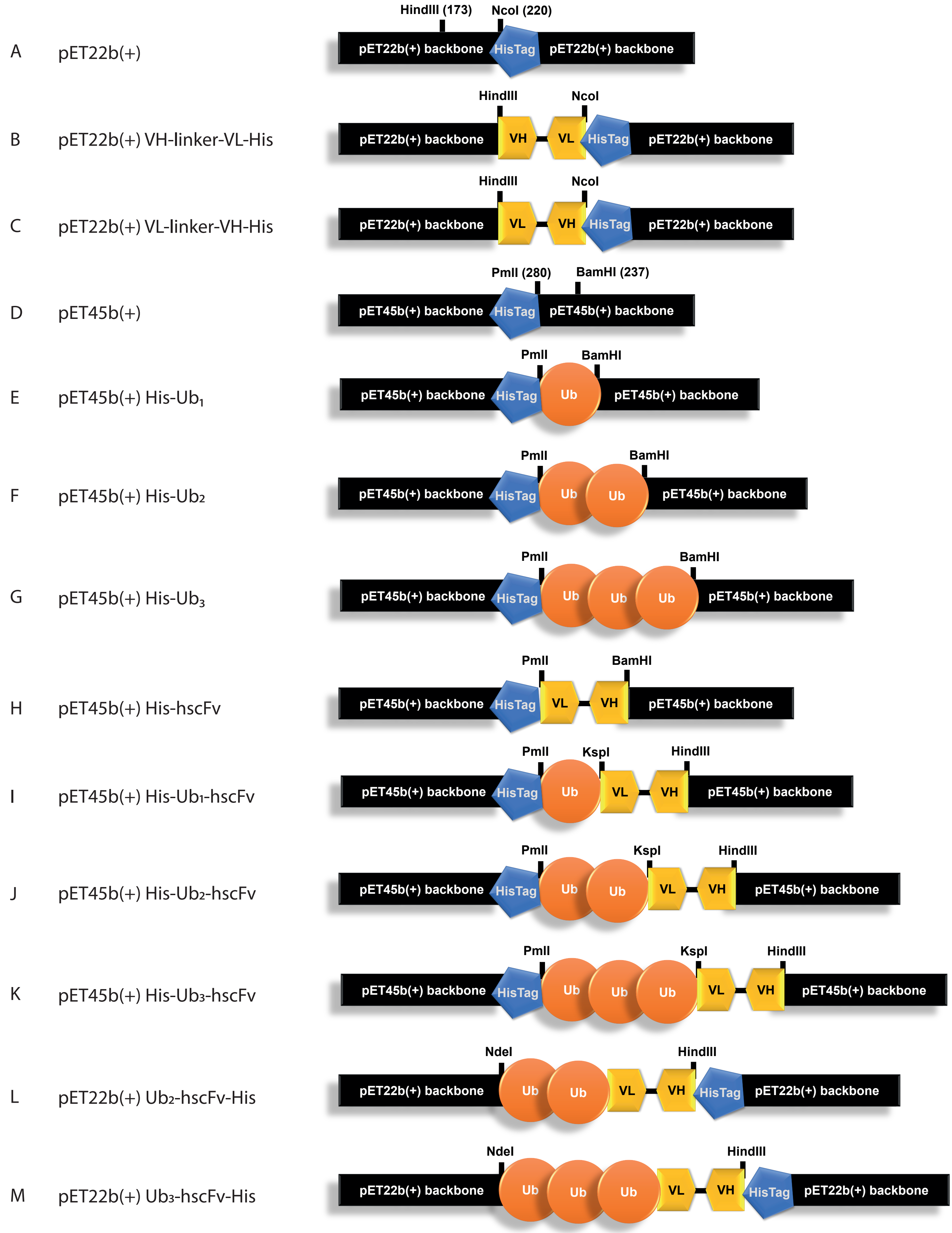

S1 Fig. Vectors and constructs used in this study. Summarizing list of all the vectors used (A and D) and constructs elaborated and produced in sequence. The VL-linker-VH and VH-linker-VL constructs in pET22b(+) vector were created to assess the best orientation (B and C). The VL-linker-VH orientation was the most soluble, hence it was chosen for the next studies and was denominated hscFv. The coding sequence for one or more ubiquitin (Ub) monomers was inserted in pET45b(+) (E, F and G) and then the hscFv was subcloned in pET45b(+) vector empty or already cloned with Ub monomer/s (H, I, J and K). His-Ub_2-hscFv and His-Ub_3-hscFv proteins could not be purified either from soluble fraction or from inclusion bodies under denaturing conditions. This was probably due to the ubiquitin hindrance that impairs the binding to the column during affinity chromatography. Therefore, the constructs Ub_2-hscFv and Ub_3-hscFv were cloned in pET22b(+) producing the Ub_2-hscFv-His and Ub3-hscFv-His that were properly purified and tested in ELISA (L and M). Ub_2-hscFv-His (L) had a better IC_{50} and was used for the *in vitro* tests with the final name of hscFv.
