# Supplementary material for: Development and in vitro characterization of a humanized scFv against fungal infections
Source: PLoS One. 2022 Oct 31;17(10):e0276786. doi: 10.1371/journal.pone.0276786 (PMC9621433; doi:10.1371/journal.pone.0276786)
Supplement: S2 Fig — A. VH-linker-VL orientation, B. VL-linker-VH orientation. MRK: protein markers (kDa); NI: not induced; 1/2/3h: hours after induction; Soluble: soluble fractions of protein; IBs: inclusion bodies, hence insoluble fractions of proteins. Arrows indicate the position of the hscFv recombinant proteins. (PDF) [file pone.0276786.s002.pdf]

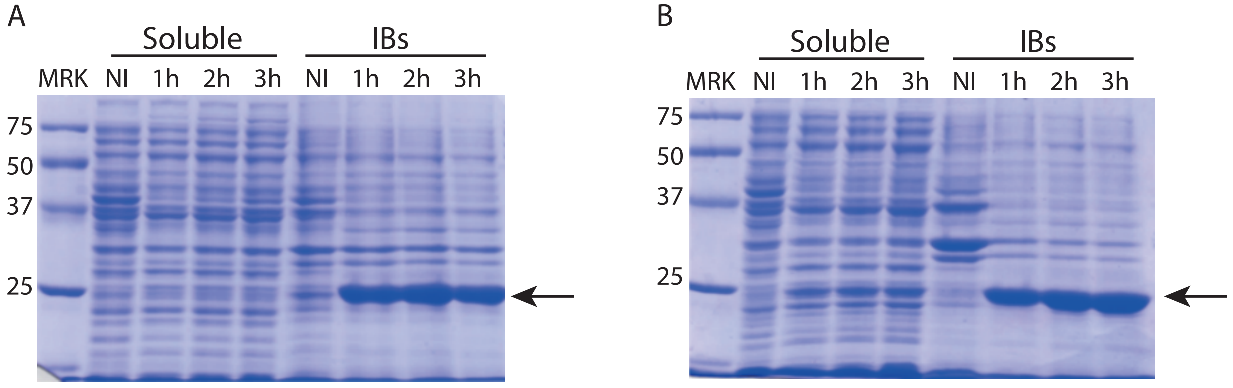

S2 Fig. SDS-PAGE of hscFvs' expressions. A. VH-linker-VL orientation, B. VL-linker-VH orientation.
MRK: protein markers (kDa); NI: not induced; 1/2/3h: hours after induction;
Soluble: soluble fractions of proteins; IBs: inclusion bodies, hence insoluble fractions of proteins.
Arrows indicate the position of the hscFv recombinant proteins.
