# Supplementary material for: Development and in vitro characterization of a humanized scFv against fungal infections
Source: PLoS One. 2022 Oct 31;17(10):e0276786. doi: 10.1371/journal.pone.0276786 (PMC9621433; doi:10.1371/journal.pone.0276786)
Supplement: S3 Fig — A. His-hscFv; B. His-Ub1-hscFv; C. His-Ub2-hscFv; D. His-Ub3-hscFv. Arrows indicate the position of the hscFv recombinant proteins. (PDF) [file pone.0276786.s003.pdf]

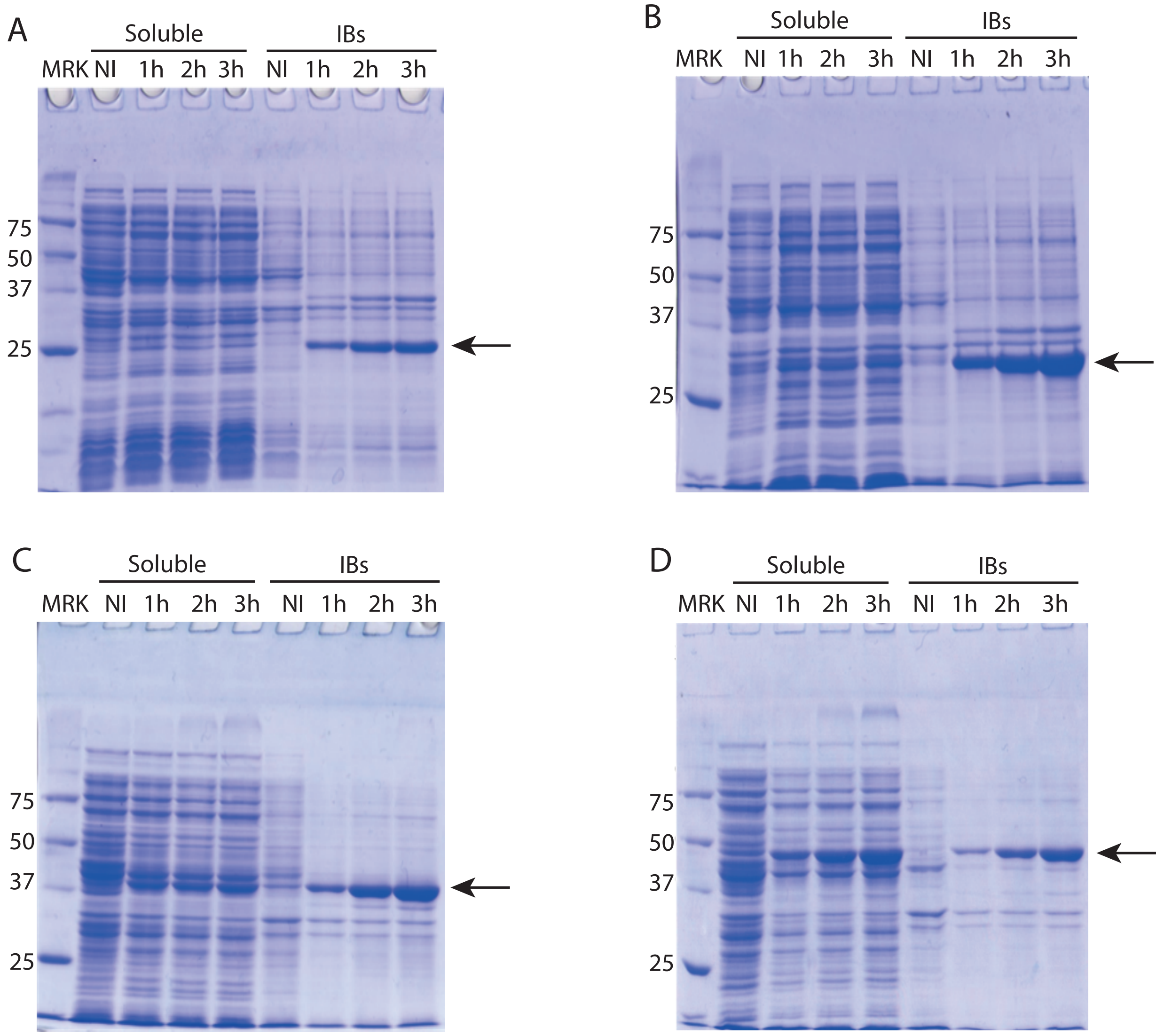

S3 Fig. SDS-PAGE expression analysis of hscFv in pET45b(+). A. His-hscFv; B. His-Ub_1-hscFv; C. His-Ub_2-hscFv; D. His-Ub_3-hscFv. Arrows indicate the position of the hscFv recombinant proteins.
