# Supplementary material for: Development and in vitro characterization of a humanized scFv against fungal infections
Source: PLoS One. 2022 Oct 31;17(10):e0276786. doi: 10.1371/journal.pone.0276786 (PMC9621433; doi:10.1371/journal.pone.0276786)
Supplement: S4 Fig — A. His-Ub2-hscFv; B. His-Ub3-hscFv. MRK:protein marker (kDa); NI: not induced; C. sample loaded into the column; FT: flow-through. Arrows indicate the position of the hscFv recombinant proteins. (PDF) [file pone.0276786.s004.pdf]

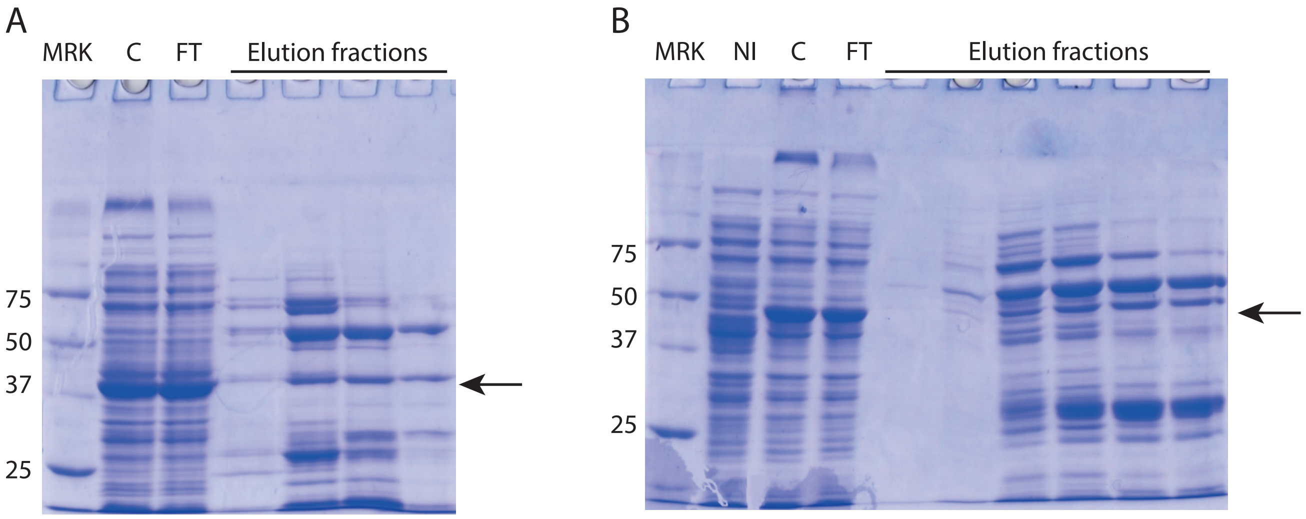

S4 Fig. Purification of Ub fusion hscFv proteins from the soluble fraction.
A. His-Ub_2-hscFv; B. His-Ub_3-hscFv. MRK: protein marker (kDa); NI: not induced; C. sample loaded into the column; FT: flow-through. Arrows indicate the position of the hscFv recombinant proteins.
