# Supplementary material for: Development and in vitro characterization of a humanized scFv against fungal infections
Source: PLoS One. 2022 Oct 31;17(10):e0276786. doi: 10.1371/journal.pone.0276786 (PMC9621433; doi:10.1371/journal.pone.0276786)
Supplement: S5 Fig — MRK: protein marker (kDa); NI: not induced; C: sample loaded into the column; FT: flow-through. Arrow indicates the position of the hscFv recombinant protein. (PDF) [file pone.0276786.s005.pdf]

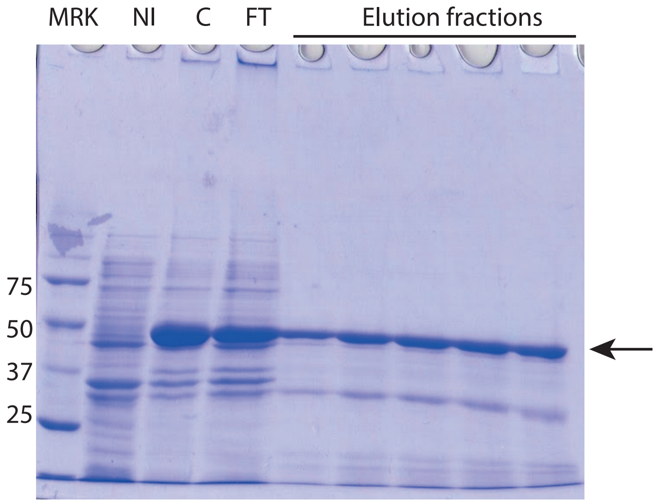

S5 Fig. Purification of His-Ub$_3$-hscFv from inclusion bodies.
MRK: protein marker (kDa); NI: not induced; C: sample loaded into the column; FT: flow-through. Arrow indicates the position of the hscFv recombinant protein.
