# Supplementary material for: Development and in vitro characterization of a humanized scFv against fungal infections
Source: PLoS One. 2022 Oct 31;17(10):e0276786. doi: 10.1371/journal.pone.0276786 (PMC9621433; doi:10.1371/journal.pone.0276786)
Supplement: S6 Fig — SDS-PAGE analysis of Ub2-hscFv-His (A) and Ub3-hscFv-His (B). MRK: protein marker (kDa); NI: not induced; 1/2/3h: hours after induction; Soluble: soluble fractions of protein; IBs: inclusion bodies, hence insoluble fractions of proteins. Arrows indicate the position of the hscFv recombinant protein. (PDF) [file pone.0276786.s006.pdf]

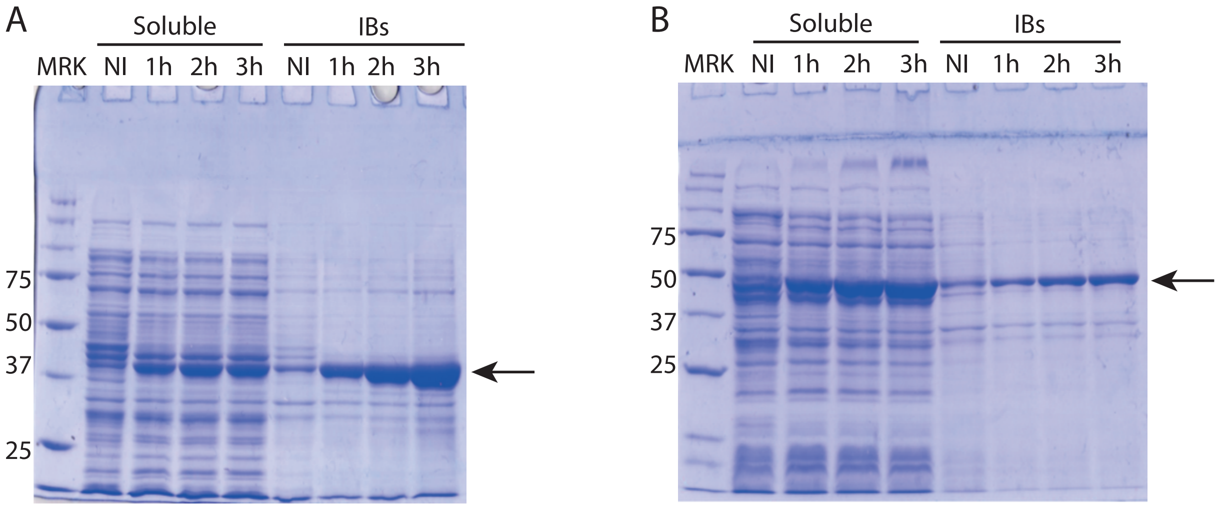

S6 Fig. SDS-PAGE analysis of Ub_2-hscFv-His (A) and Ub_3-hscFv-His (B).
MRK: protein markers (kDa); NI: not induced; 1/2/3h: hours after induction;
Solube: soluble fractions of proteins; IBs: inclusion bodies, hence insoluble fractions of proteins.
Arrows indicate the position of the hscFv recombinant proteins.
