# Supplementary material for: Development and in vitro characterization of a humanized scFv against fungal infections
Source: PLoS One. 2022 Oct 31;17(10):e0276786. doi: 10.1371/journal.pone.0276786 (PMC9621433; doi:10.1371/journal.pone.0276786)
Supplement: S7 Fig — Purification of Ub2-hscFv-His (A) and Ub3-hscFv-His (B) from the soluble fraction. MRK: protein marker (kDa); C: sample loaded into the column; FT: flow-through. (PDF) [file pone.0276786.s007.pdf]

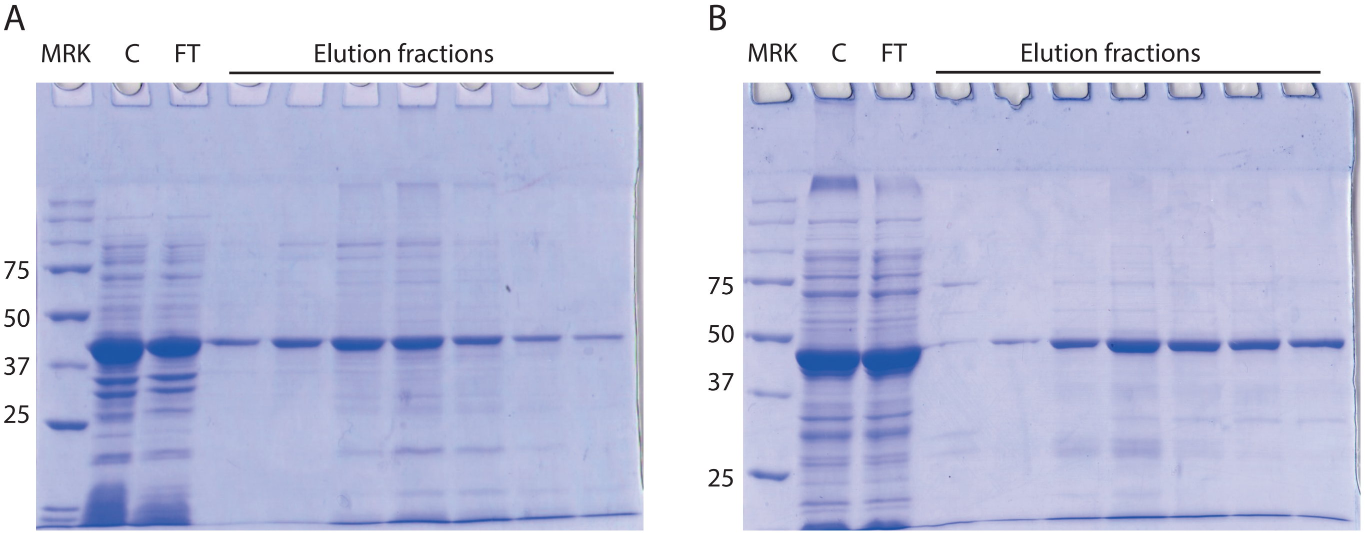

S7 Fig. Purification or Ub_2-hscFv-His (A) and Ub_3-hscFv-His (B) from the soluble fraction.
MRK: protein marker (kDa); C: sample loaded into the column; FT: flow-through.
