# Supplementary material for: Development and in vitro characterization of a humanized scFv against fungal infections
Source: PLoS One. 2022 Oct 31;17(10):e0276786. doi: 10.1371/journal.pone.0276786 (PMC9621433; doi:10.1371/journal.pone.0276786)
Supplement: S8 Fig — A. Single passage purification of Ub2-hscFv-His at pH 8.5, step at 100 mM of imidazole and gradient to 250 mM of imidazole. B-C. Double passage purification of Ub2-hscFv-His: negative passage in Q Sepharose FF at pH 7.4, elution with NaCl (B) and positive passage in IMAC at pH 8.5, step at 100 mM of imidazole and gradient to 250 mM of imidazole (C). MRK: protein marker (kDa); C: sample loaded onto the column; FT: flow-through. (PDF) [file pone.0276786.s008.pdf]

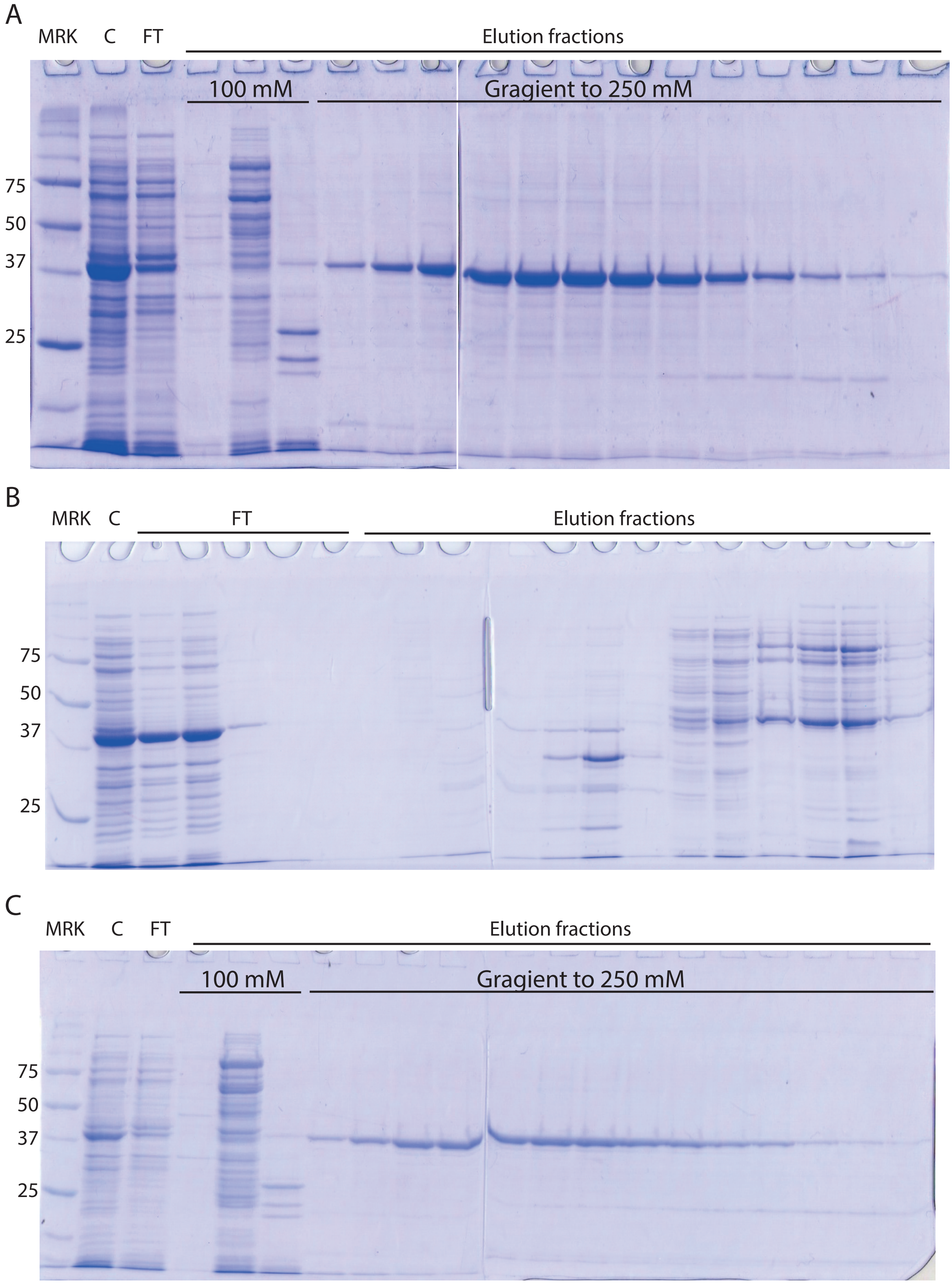

S8 Fig. Passages of the optimized purification protocol. A. Single passage purification of Ub_2-hscFv-His at pH 8.5, step at 100 mM of imidazole and gradient to 250 mM of imidazole. B-C. Double passage purification of Ub_2-hscFv-His: negative passage in Q Sepharose FF at pH 7.4, elution with NaCl (B) and positive passage in IMAC at pH 8.5, step at 100 mM of imidazole and gradient to 250 mM of imidazole (C). MRK: protein marker (kDa); C: sample loaded into the column; FT: flow-through.
