# Supplementary material for: Development and in vitro characterization of a humanized scFv against fungal infections
Source: PLoS One. 2022 Oct 31;17(10):e0276786. doi: 10.1371/journal.pone.0276786 (PMC9621433; doi:10.1371/journal.pone.0276786)
Supplement: S9 Fig — hscFv was stored at different temperatures; 37°C, 4°C, -20°C, -80°C, and for different timepoints. The solubility and the integrity of the recombinant protein was evaluated through SDS-PAGE and western immunoblotting. The samples were loaded in 10% polyacrylamide gels and stained with Brilliant Blue Coomassie R-250 or electroblotted onto a nitrocellulose membrane for protein detection. (PDF) [file pone.0276786.s009.pdf]

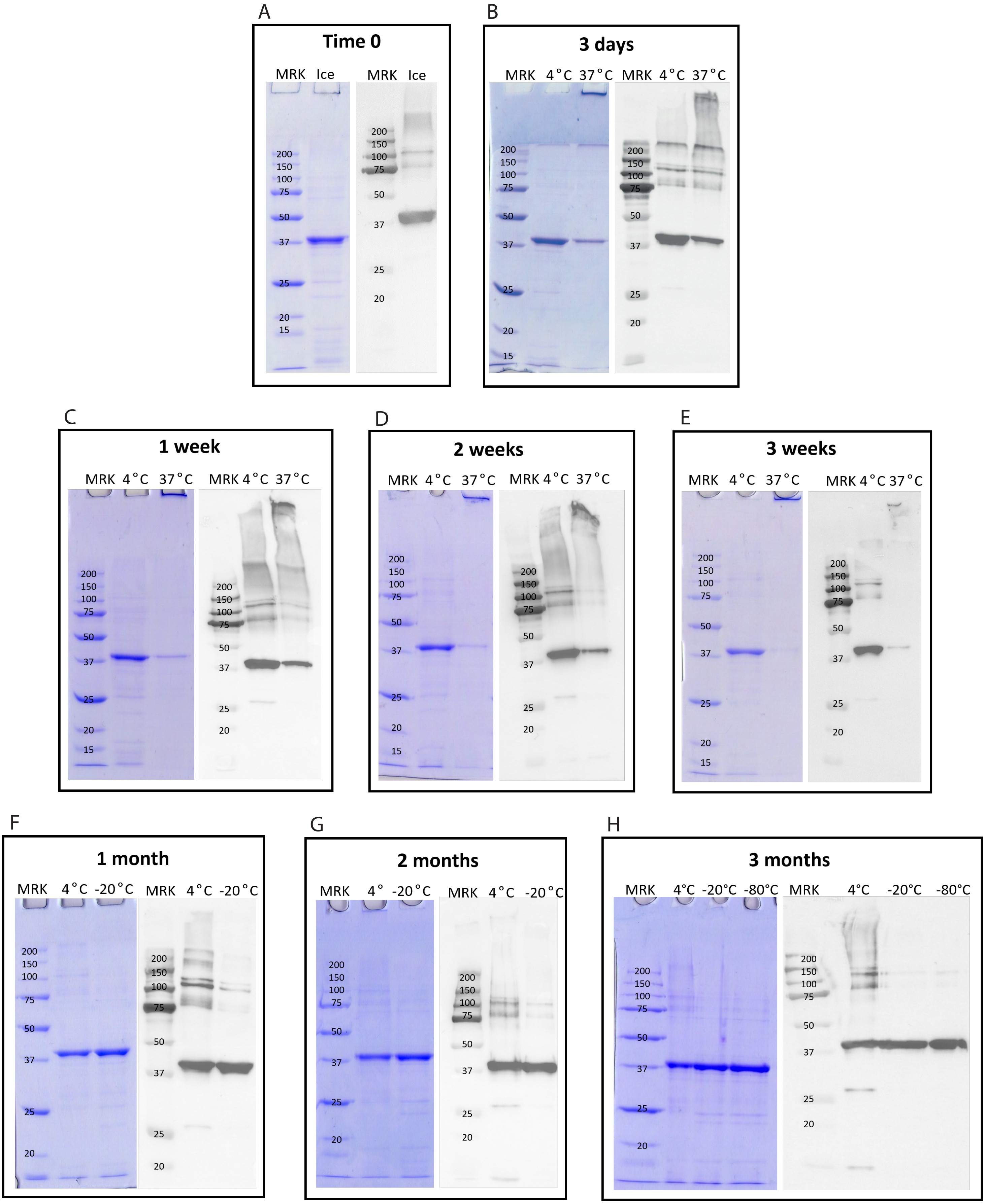

S9 Fig. Stability test - Evaluation of the formation of aggregates after storing the hscFv at different temperatures and for different timepoints. HscFv was stored at different temperatures; 37 °C, 4 °C, -20 °C, -80 °C, and for different timepoints. The solubility and the integrity of the recombinant protein was evaluated through SDS-PAGE and western immunoblotting. The samples were loaded in 10% polyacrylamide gels and stained with Brilliant Blue Coomassie R-250 or electroblotted onto a nitrocellulose membrane for protein detection.
