# Supplementary material for: Development and in vitro characterization of a humanized scFv against fungal infections
Source: PLoS One. 2022 Oct 31;17(10):e0276786. doi: 10.1371/journal.pone.0276786 (PMC9621433; doi:10.1371/journal.pone.0276786)
Supplement: S10 Fig — From a FSC vs SSC contour plot (depicting cell physical characteristics) specific gates surround green and blue events. On the left C. auris cells: in red the entire sample population; in green the subpopulation of the unbudded cells; in blue the subpopulation of the budded cells. On the right C. albicans cells: in red the entire sample population; in green the subpopulation of the cells in yeast form; in blue the subpopulation of the cells in hyphal form. (PDF) [file pone.0276786.s010.pdf]

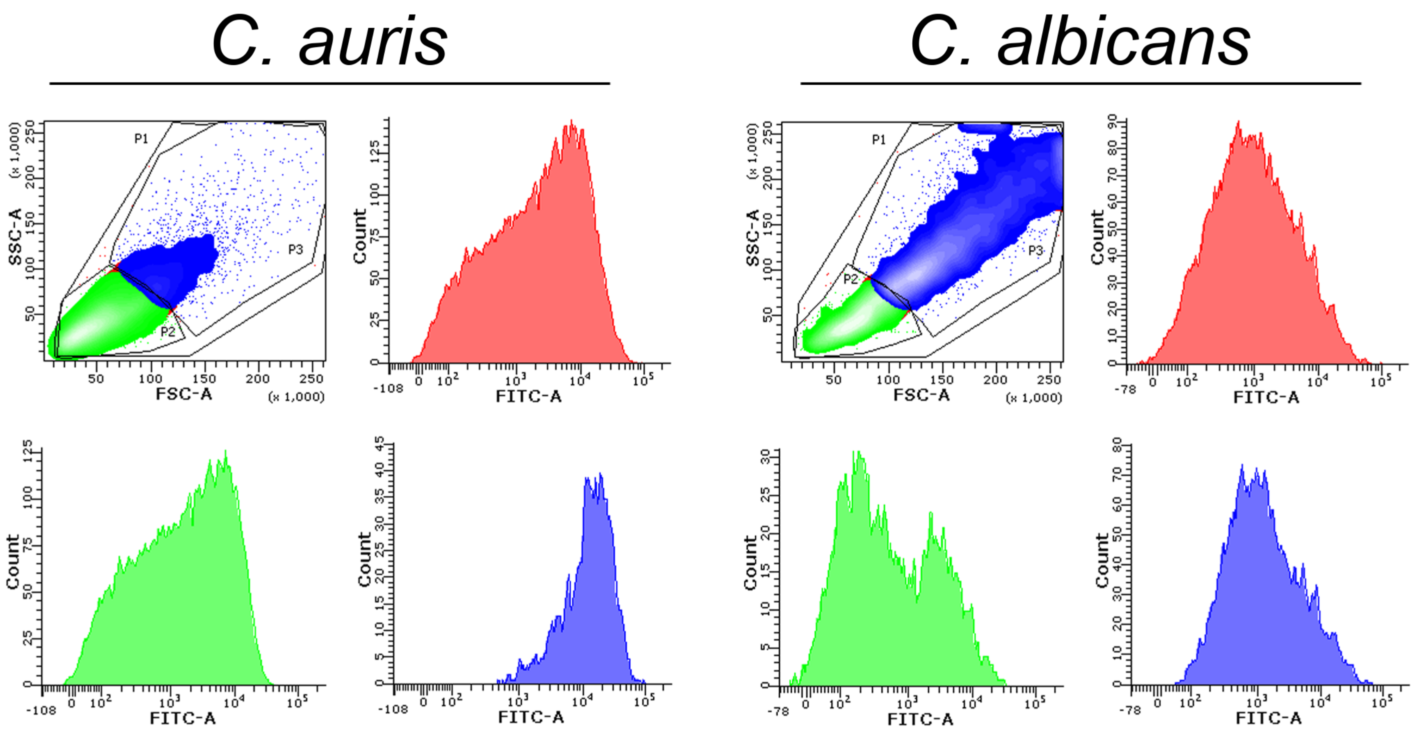

S10 Fig. Flow cytometric analysis of MFI of *C. auris* and *C. albicans* cells treated with hscFv.
From a FSC vs SSC contour plot (depicting cell physical characteristics) specific gates surround green and blue events. On the left *C. auris* cells: in red the entire sample population; in green the subpopulation of the unbudded cells; in blue the subpopulation of the budded cells.
On the right *C. albicans* cells: in red the entire sample population; in green the subpopulation of the cells in yeast form; in blue the subpopulation of the cells in hyphal form.
