# Supplementary material for: Development and in vitro characterization of a humanized scFv against fungal infections
Source: PLoS One. 2022 Oct 31;17(10):e0276786. doi: 10.1371/journal.pone.0276786 (PMC9621433; doi:10.1371/journal.pone.0276786)
Supplement: S11 Fig — Caspofungin was tested alone and in combination with different concentrations of hscFv against C. auris. The histograms represent the fungal growth as determined by measuring the Abs at 405 nm. The mean ± SD of the Abs read after 24 (A) and 48 (B) hours were obtained from three independent experiments performed in triplicate. The growth of untreated cells was used as control. With MIC50 we considered the lowest concentrations that inhibit 50% of the fungal growth compared to the drug-free control (the red line threshold). (PDF) [file pone.0276786.s011.pdf]

A

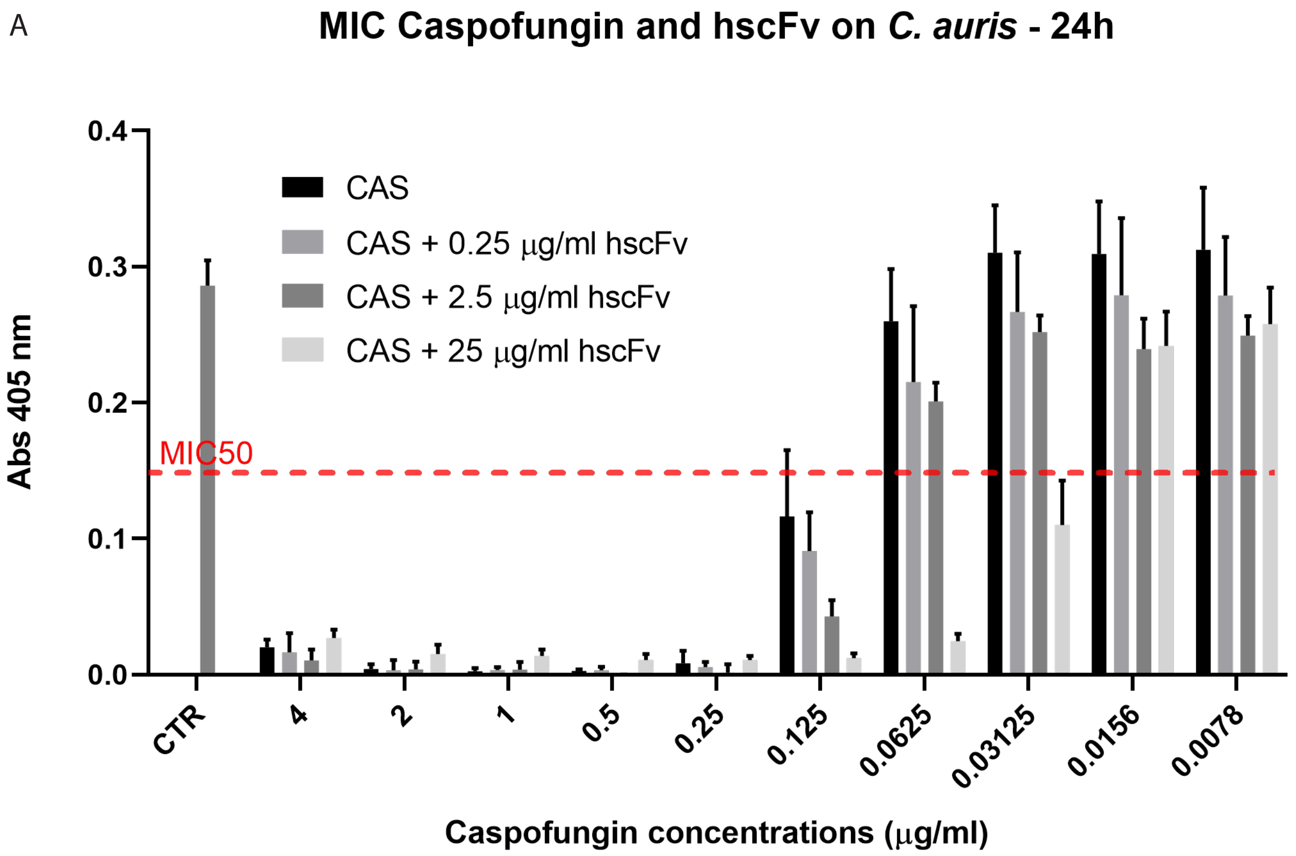


B

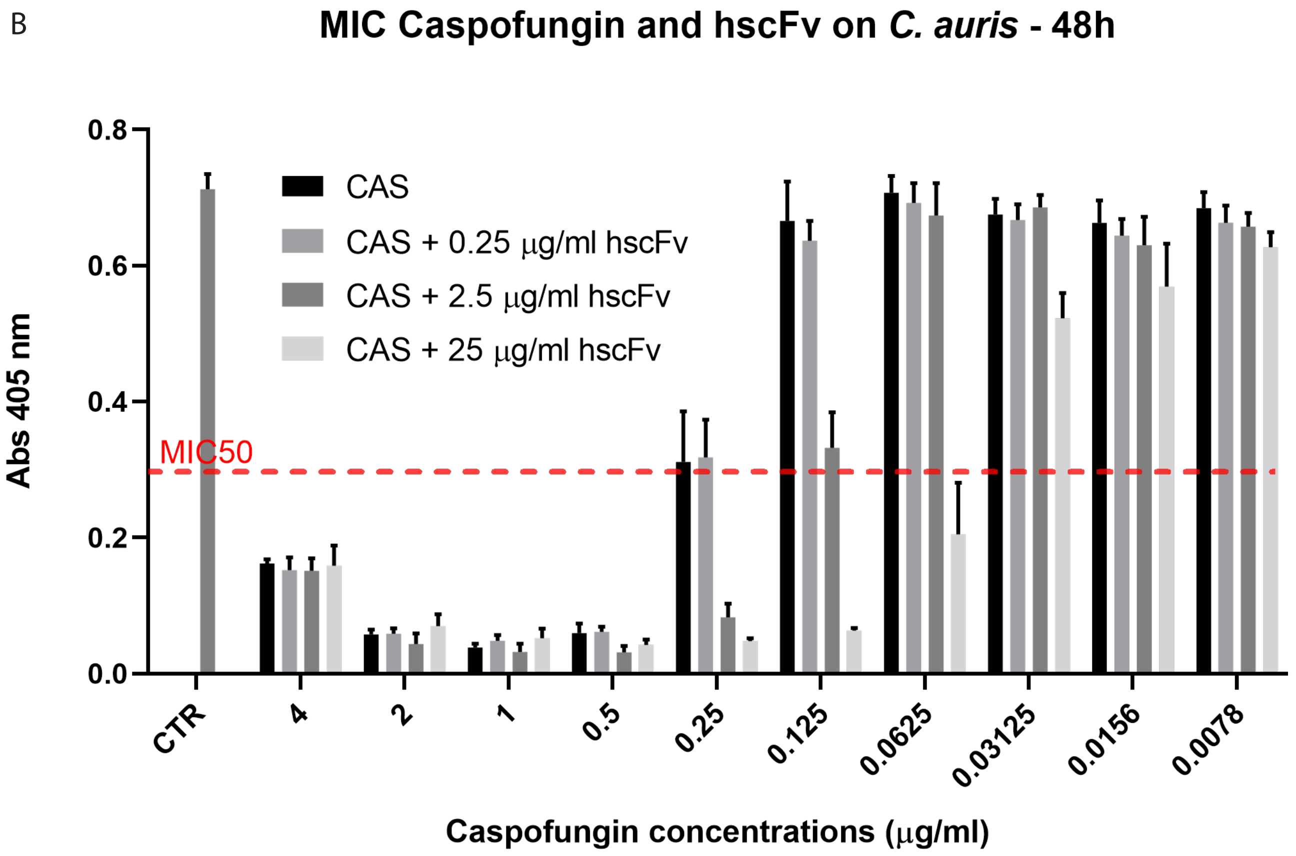


**S11 Fig. MIC assay of caspofungin (CAS) alone and in combination with hscFv.** Caspofungin was tested alone and in combination with different concentrations of hscFv against *C. auris*. The histograms represent the fungal growth as determined by measuring the Abs at 405 nm. The mean ± SD of the Abs read after 24 (A) and 48 (B) hours were obtained from three independent experiments performed in triplicate. The growth of untreated cells was used as control. With MIC50 we considered the lowest concentrations that inhibit 50% of the fungal growth compared to the drug-free control (the red line threshold).
