# Supplementary material for: Development and in vitro characterization of a humanized scFv against fungal infections
Source: PLoS One. 2022 Oct 31;17(10):e0276786. doi: 10.1371/journal.pone.0276786 (PMC9621433; doi:10.1371/journal.pone.0276786)

Fig. 2

(X: sample 3 after dialysis and concentration.)

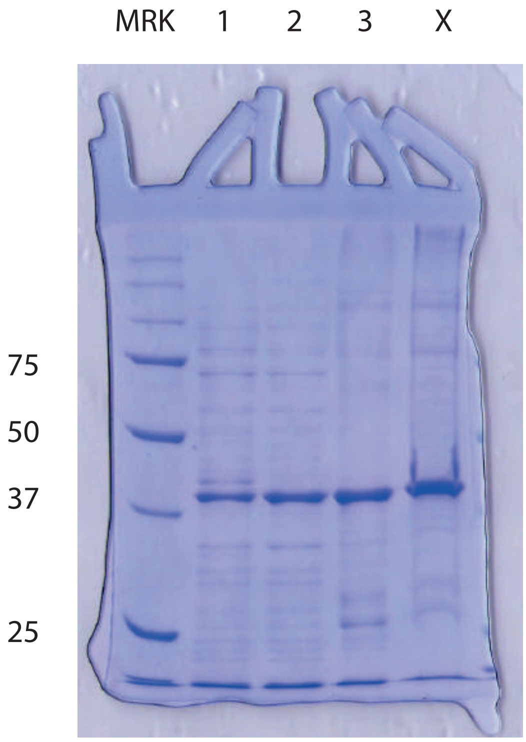

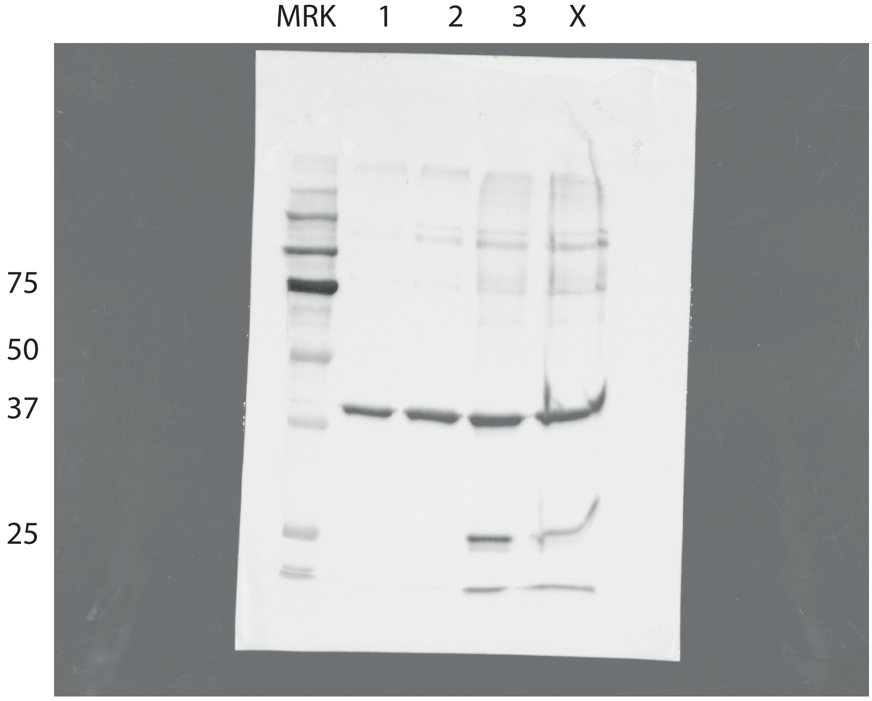

S2 Fig.

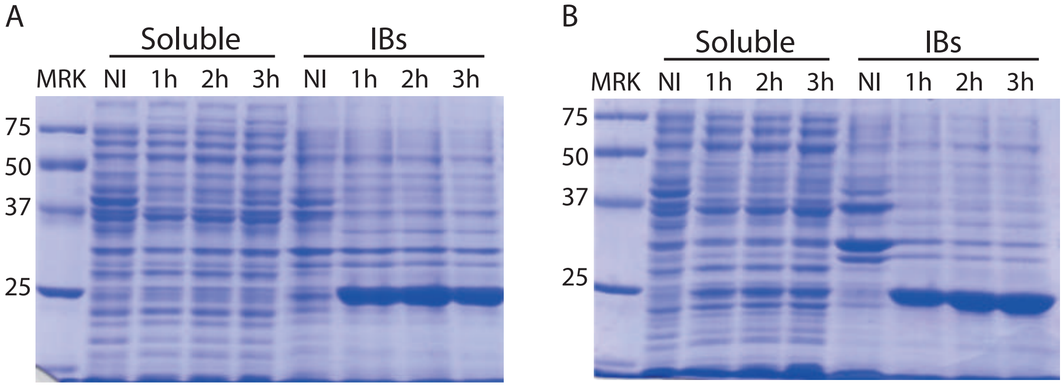
A
Soluble
IBs
MRK NI 1h 2h 3h NI 1h 2h 3h
75
50
37
25
B
Soluble
IBs
MRK NI 1h 2h 3h NI 1h 2h 3h
75
50
37
25

S3 Fig.

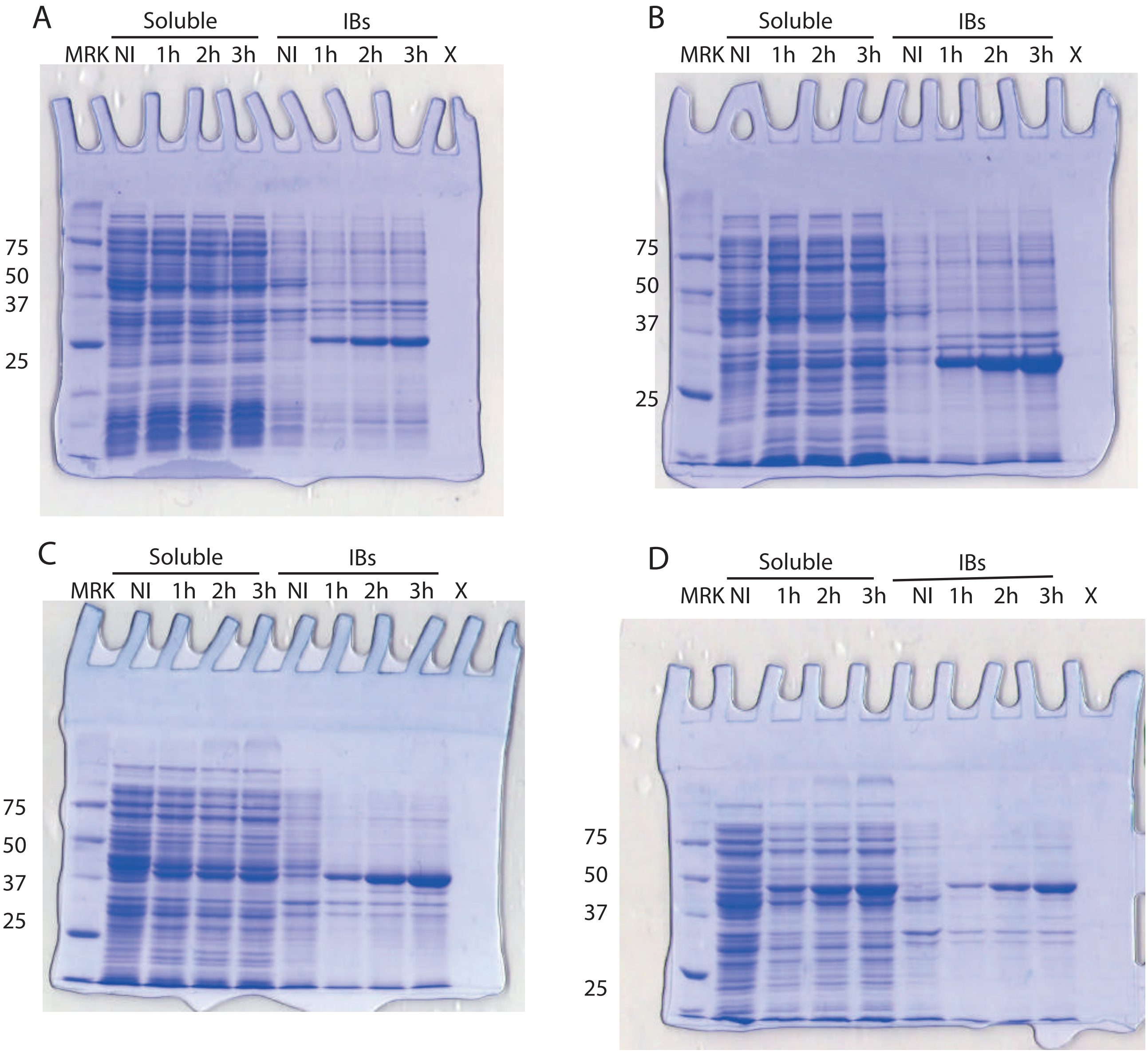
A
Soluble
IBs
MRK NI 1h 2h 3h NI 1h 2h 3h X
75
50
37
25
B
Soluble
IBs
MRK NI 1h 2h 3h NI 1h 2h 3h X
75
50
37
25
C
Soluble
IBs
MRK NI 1h 2h 3h NI 1h 2h 3h X
75
50
37
25
D
Soluble
IBs
MRK NI 1h 2h 3h NI 1h 2h 3h X
75
50
37
25

S4 Fig.

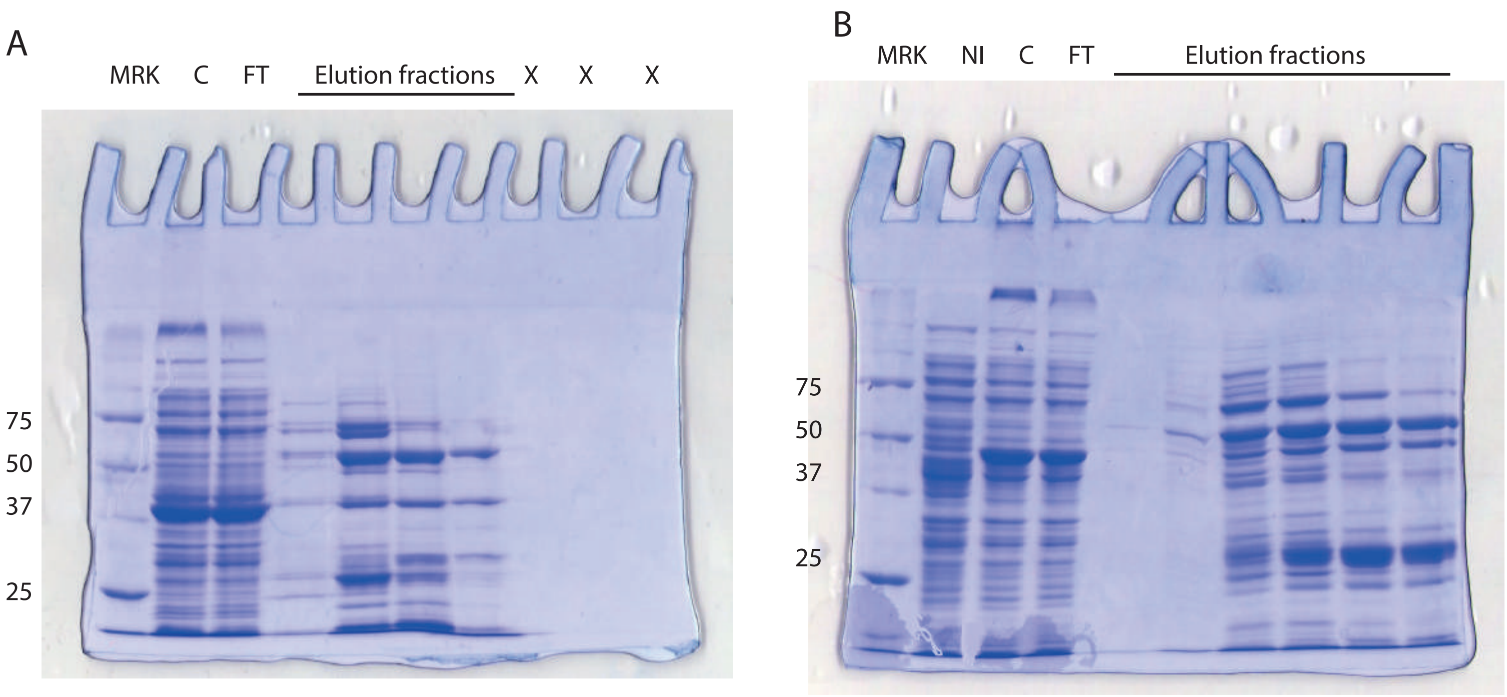
A
MRK
C
FT
Elution fractions
X
X
X
75
50
37
25
B
MRK
NI
C
FT
Elution fractions
75
50
37
25

S5 Fig.

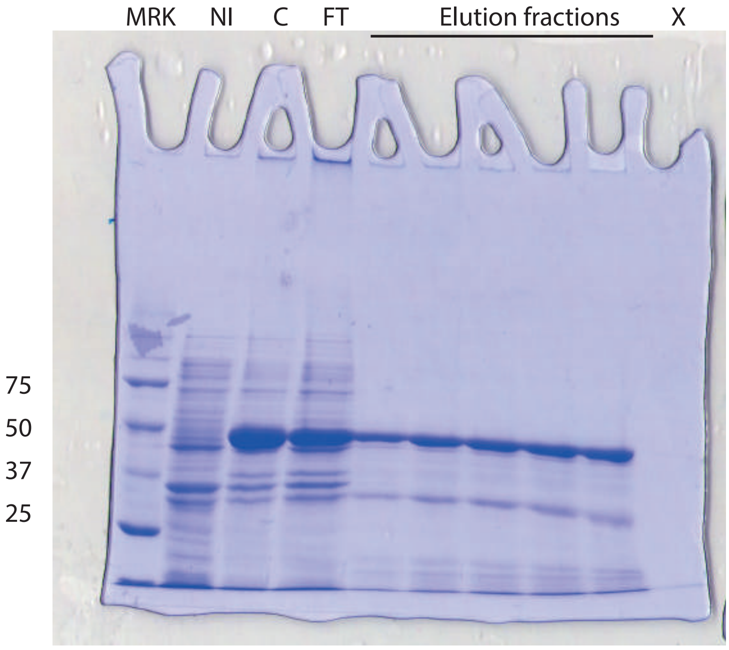
MRK
NI
C
FT
Elution fractions
X
75
50
37
25

S6 Fig.

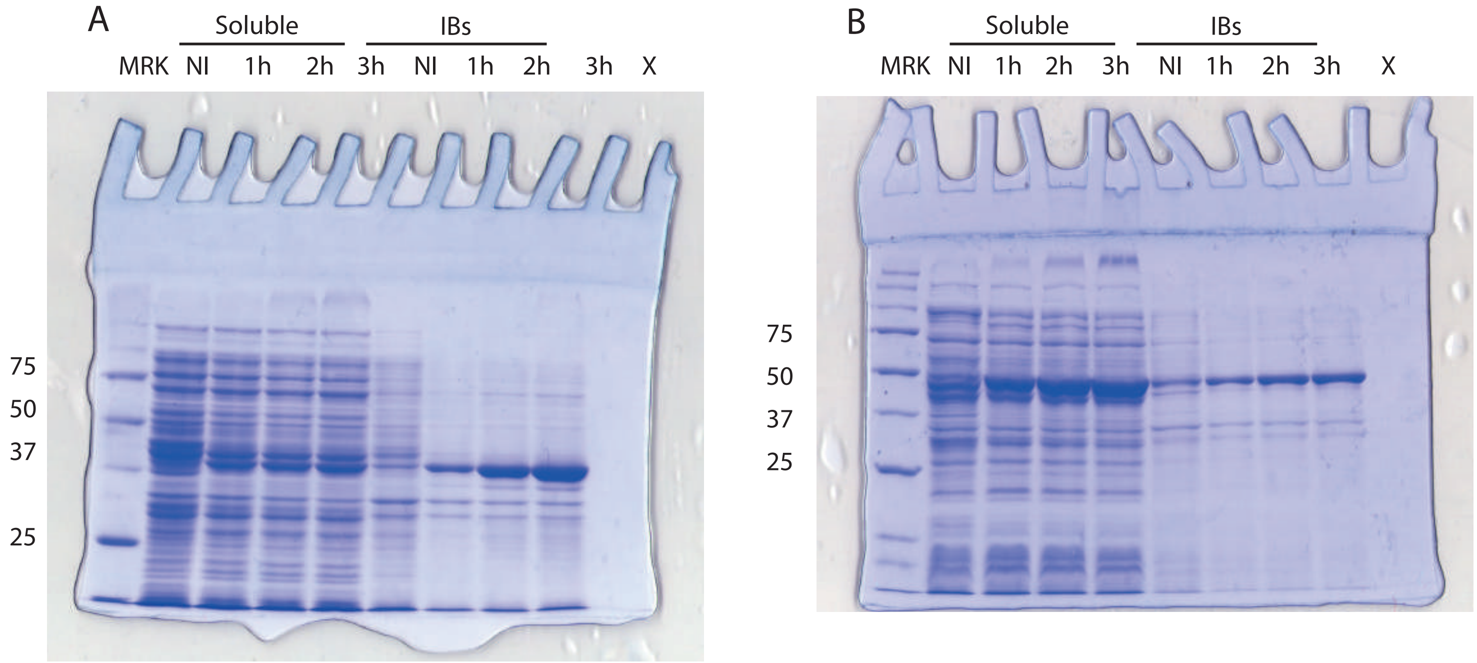
A
Soluble
IBs
MRK NI 1h 2h 3h NI 1h 2h 3h X
75
50
37
25
B
Soluble
IBs
MRK NI 1h 2h 3h NI 1h 2h 3h X
75
50
37
25

S7 Fig.

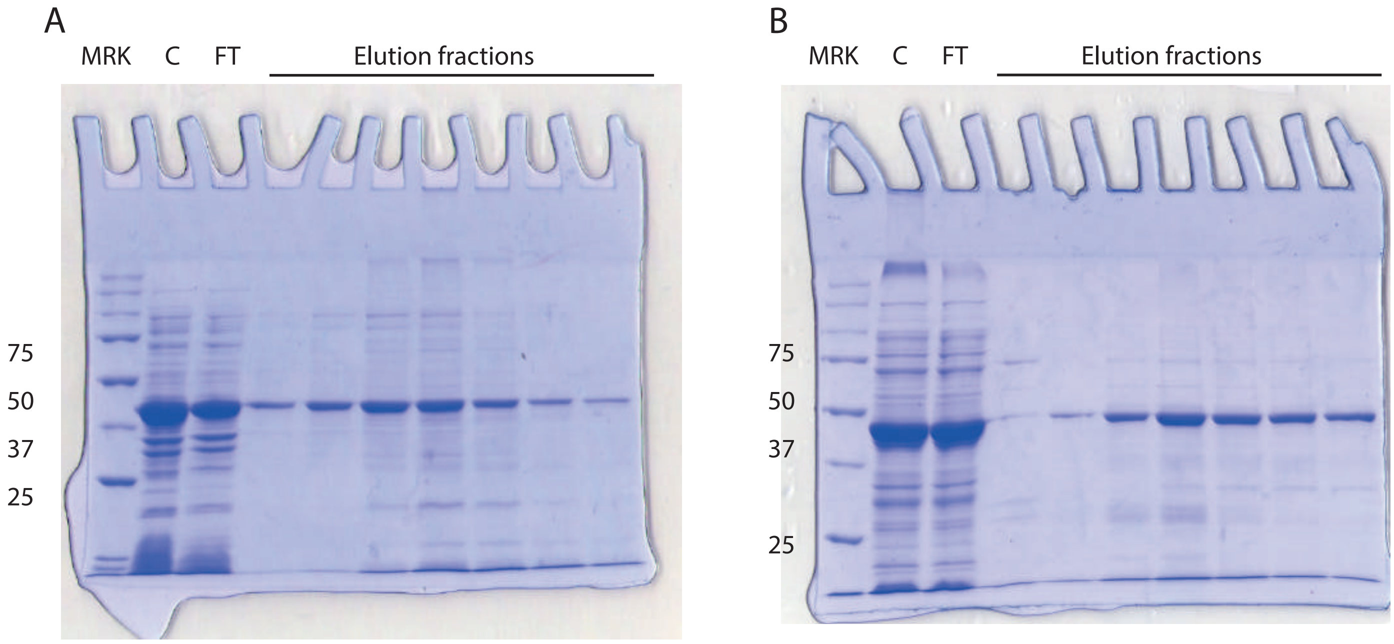
A
MRK C FT
Elution fractions
75
50
37
25
B
MRK C FT
Elution fractions
75
50
37
25

S8 Fig.

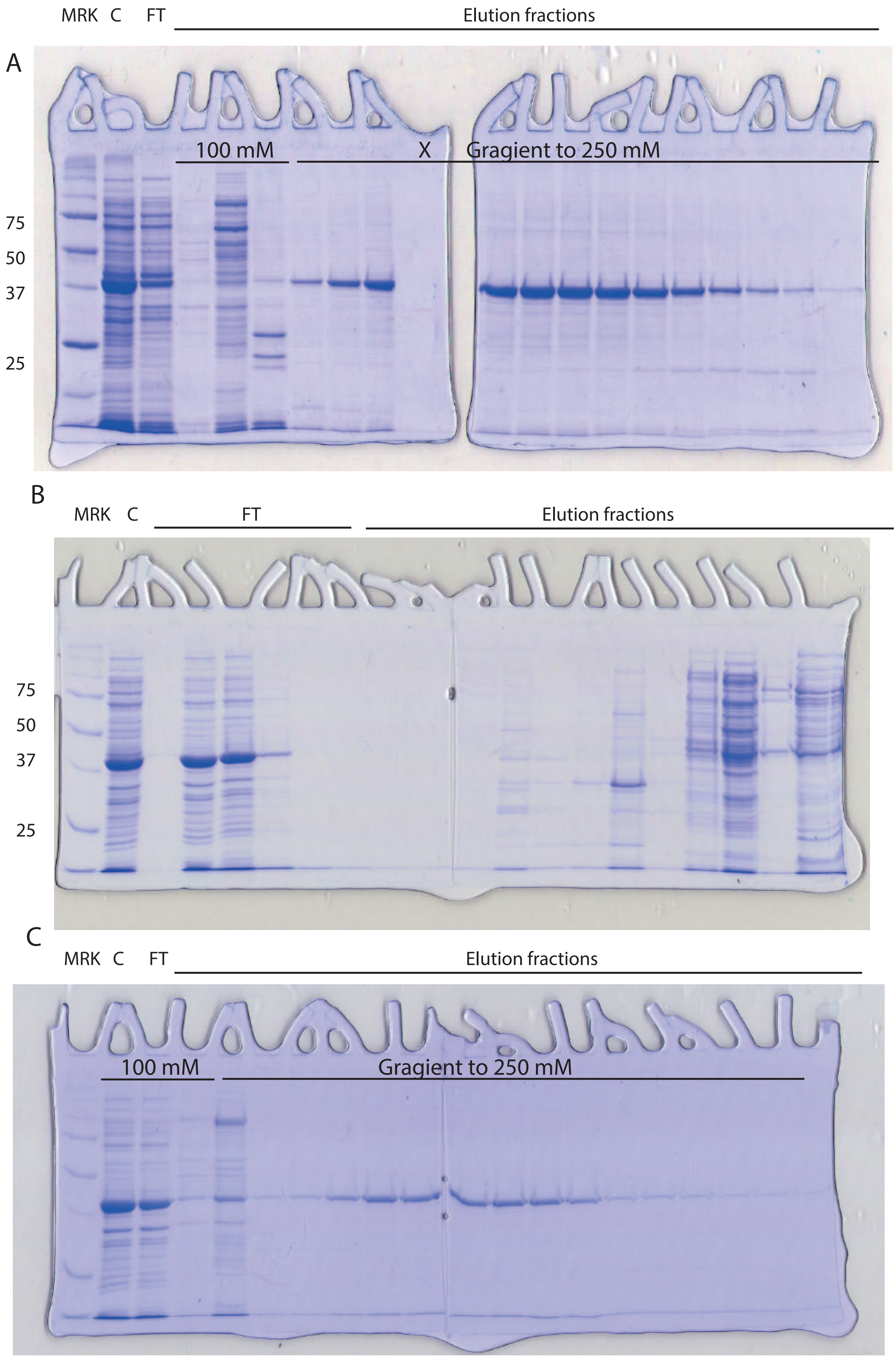
MRK C FT
Elution fractions
A
100 mM
X
Gragient to 250 mM
75
50
37
25
B
MRK C
FT
Elution fractions
75
50
37
25
C
MRK C FT
Elution fractions
100 mM
Gragient to 250 mM
75
50
37
25

S9 Fig.

A

Time 0

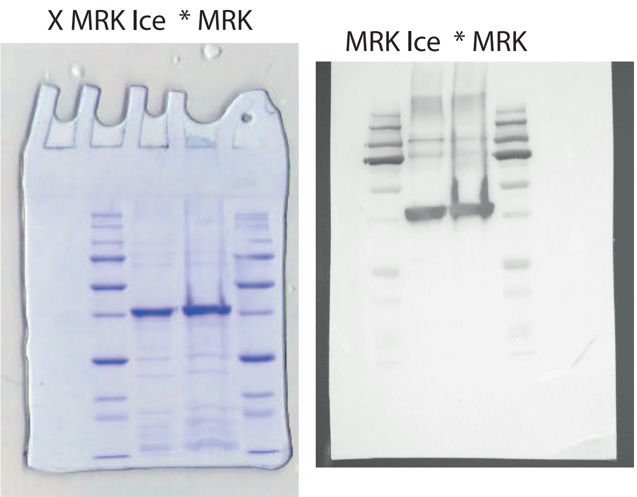

* is the same sample of "Ice", just a different concentration.

B

3 days

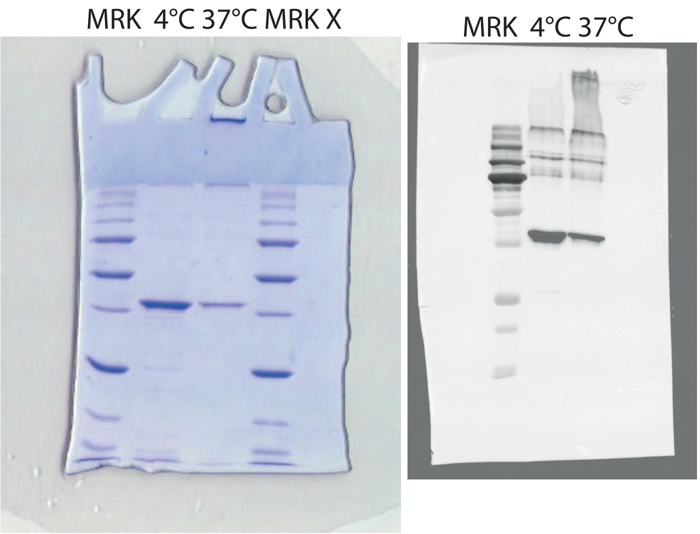

C

1 week

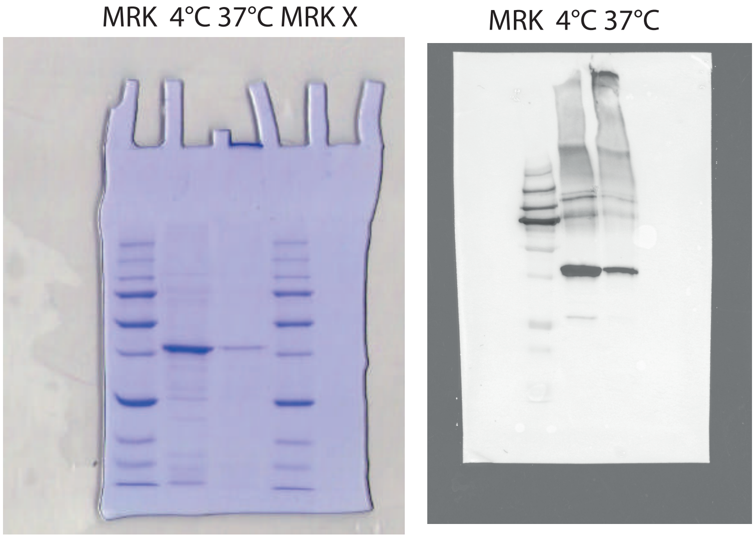

D

2 weeks

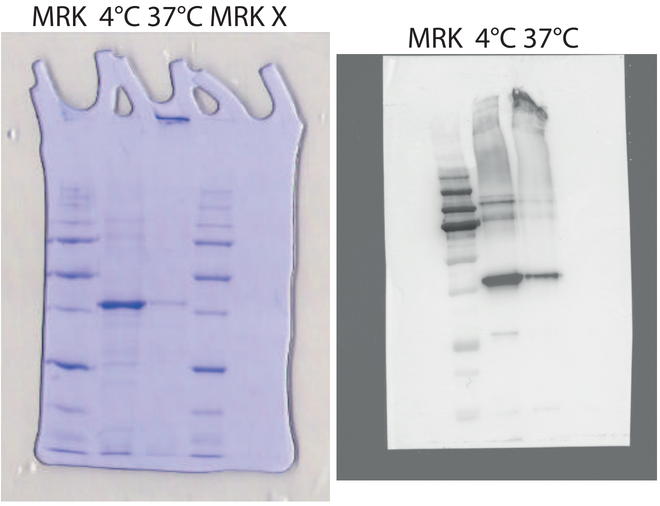

E

3 weeks

MRK 4°C 37°C X X X

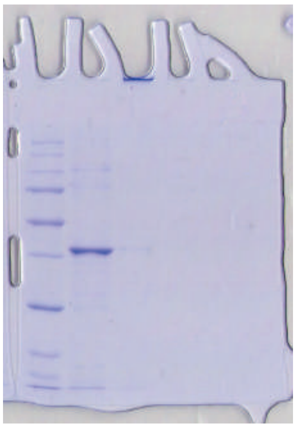

MRK 4°C 37°C

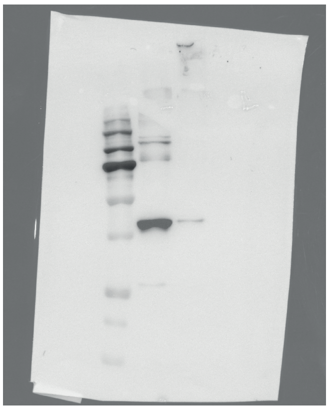

F

1 month

MRK 4°C -20°C X X

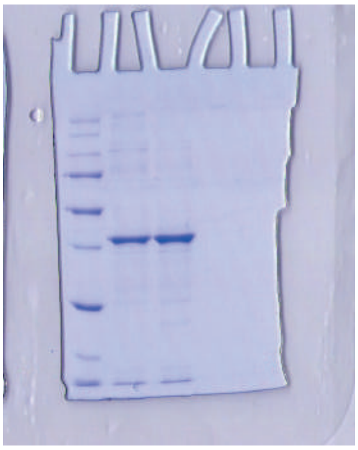

MRK 4°C -20°C

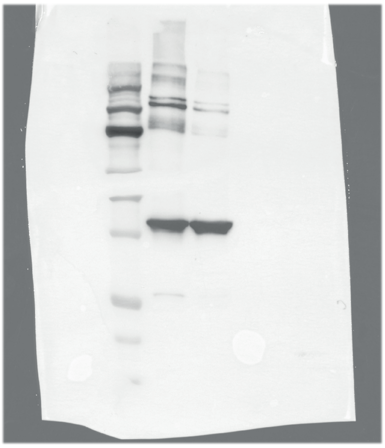

G

2 months

MRK 4°C -20°C MRK X

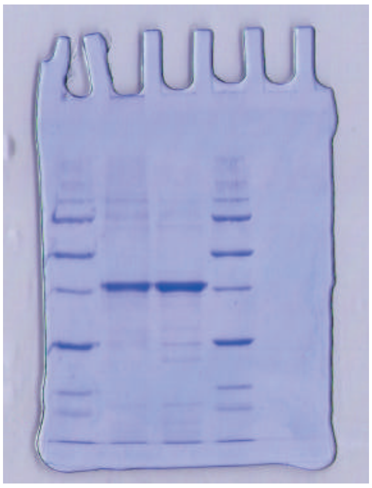

MRK 4°C -20°C MRK

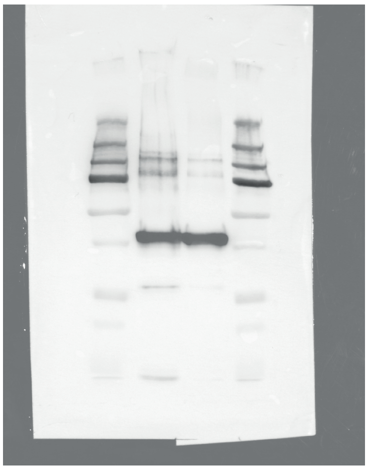

H

3 months

MRK 4°C -20°C -80°C X

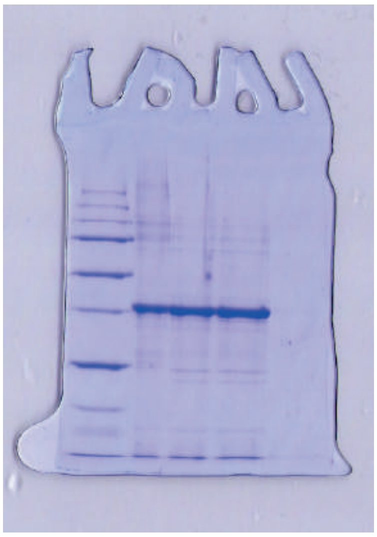

MRK 4°C -20°C -80°C

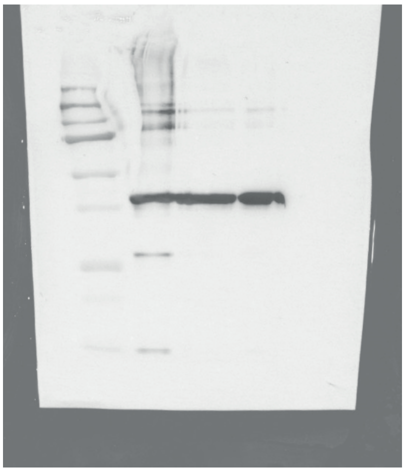

Supplement: S1 Raw images — (PDF) [file pone.0276786.s013.pdf]
